# Synthesis of Lasofoxifene, Nafoxidine and Their Positional Isomers via the Novel Three-Component Coupling Reaction

**DOI:** 10.3390/molecules15106773

**Published:** 2010-09-28

**Authors:** Kenya Nakata, Yoshiyuki Sano, Isamu Shiina

**Affiliations:** Department of Applied Chemistry, Faculty of Science, Tokyo University of Science, 1-3 Kagurazaka, Shinjuku-ku, Tokyo 162-8601, Japan

**Keywords:** three-component coupling reaction, diversity oriented synthesis, lasofoxifene, nafoxidine, *inv*-lasofoxifene, *inv*-nafoxidine

## Abstract

A Lewis acid-mediated three-component coupling reaction was successfully applied for the synthesis of lasofoxifene (**1**), nafoxidine (**2**), and their positional isomers, *inv*-lasofoxifene (**3**) and *inv*-nafoxidine (**4**). In the presence of HfCl_4_, the desired one-pot coupling reaction among 4-pivaloyloxybenzaldehyde (**5**), cinnamyltrimethylsilane (**6**), and anisole proceeded to afford the corresponding 3,4,4-triaryl-1-butene **7** in high yield. The iodocarbocyclization of the coupling product and the successive elimination of hydrogen iodide forming the olefin part, followed by the migration of the double-bond afforded the common synthetic intermediate of lasofoxifene (**1**) and nafoxidine (**2**) via a very concise procedure. Additionally, the syntheses of their positional isomers *inv*-lasofoxifene (**3**) and *inv*-nafoxidine (**4**) were also achieved through very convenient protocols.

## 1. Introduction

Multi-component coupling reactions (MCRs) are one of the most important tools in organic synthesis for the divergent production of bioactive molecules and highly complicated natural products [1,2,3]. In the beginning, the Strecker- and the Mannich-reactions were developed as three-component coupling reactions to form the corresponding α-amino or β-amino acids, and a variety of biologically important compounds were prepared in a one-pot operation according to these useful procedures. After that, a more advanced four-segment coupling reaction was reported by Ugi *et al*. The Ugi-reaction directly forms various α-amino acid derivatives from a mixture of primary amine, aldehyde, isonitrile, and carboxylic acid in one-pot, so that it was successfully utilized in the effective syntheses of a number of natural products and pharmaceutically valuable molecules [4,5]. Recent progress of synthetic organic chemistry has further established other kinds of MCRs that provide large-size oligosaccharides using sequential stereoselective glycosylations [6,7] or bioactive heterocyclic compounds starting from the conjugated organic molecules using transition-metal catalysts [8,9].

**Figure 1:**
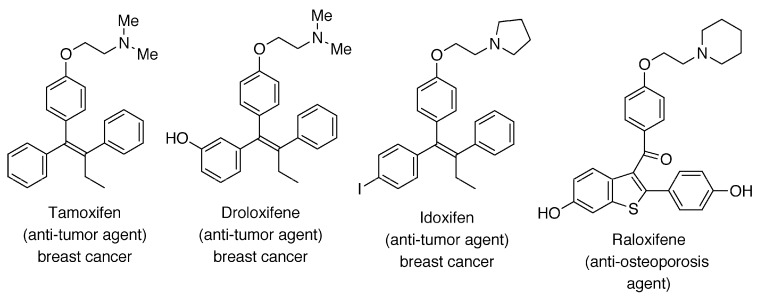
Structures of representive SERMs: tamoxifen, droloxifene, idoxifen, and raloxifene.

**Figure 2:**
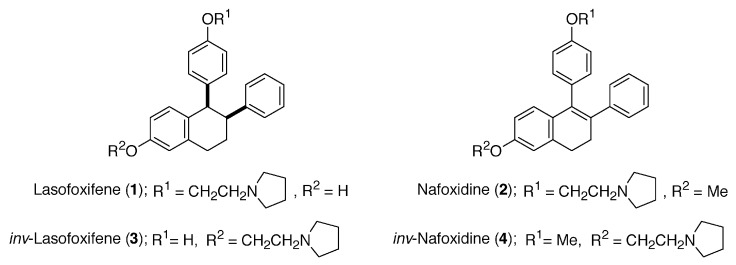
Structures of lasofoxifene (**1**) and nafoxidine (**2**) and their positional isomers (**3**) and (**4**).

We have also developed a novel and useful three-component coupling reaction among aromatic aldehydes, allylmetal reagents, and aromatic nucleophiles in the presence of a Lewis acid catalyst to afford 4,4-diaryl-1-butenes [10,11], which contain the basic skeleton of selective estrogen receptor modulators (SERMs, **Figure 1**) [12,13,14,15]. We further applied this methodology for the preparation of biologically active molecules, such as tamoxifen [16,17], droloxifene [17,18], lasofoxifene [19], nafoxidine [19], and *pseudo*-symmetric tamoxifen derivatives [20,21,22], *etc*. Related MCRs to form various substituted aromatic compounds were currently reported by other groups [23,24,25,26]. Herein, we present full results of the synthesis of lasofoxifene (**1**) and nafoxidine (**2**) as well as their positional isomers, *inv*-lasofoxifene (**3**) and *inv*-nafoxidine (**4**), to indicate further synthetic utilities of the novel three-component coupling reaction (**Figure 2**).

Lasofoxifene (**1**), discovered in 1996, is a new drug belonging to the third-generation SERMs and the current hopeful candidate for the treatment of osteoporosis, with clinical trials now in significant progress [27,28]. After observing the promising results of **1** on osteoporosis, a few improved methods for the preparation of **1** have been reported. As shown in the following examples, all protocols for the generation of lasofoxifene (**1**) utilized nafoxidine (**2**) or its derivative as a precursor of **1**. It should be noted that **2** itself is also known as a SERM [29]. First, Lednicer *et al*. reported a method for the synthesis of **2** via the dehydration reaction of the tertiary alcohol, which was obtained by the reaction of an aromatic Grignard reagent with 2-phenyl-1-tetralone generated from a chalcone derivative [30,31,32,33]. Cameron *et al*. at Pfizer, Inc., developed their original pathway to produce **1** and **2** via the Suzuki coupling between 1-aryl-2-bromo-3,4-dihydronaphthalene and phenylboronic acid [34,35]. The Chu group at Pfizer, Inc., also showed an alternative route to **1** and **2** via the intramolecular reductive coupling process of a diketone using low-valent titanium species to form the desired 1,2-diaryl-3,4-dihydronaphthalene [36]. Due to all of these synthetic methods for the preparation of **1** and **2** requiring relatively longer routes to form the desired backbone of the targeted molecules, it is desirable to develop an improved and facile protocol for providing **2** using new technologies. 

## 2. Results and Discussion

**Scheme 1:**
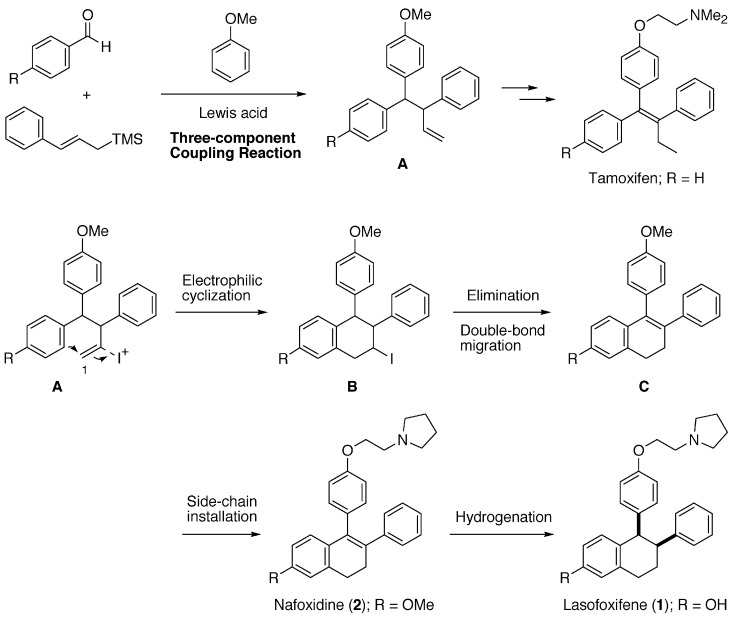
Synthetic strategy for providing lasofoxifene (**1**) and nafoxidine (**2**).

Our synthetic strategy of lasofoxifene (**1**) and nafoxidine (**2**) is outlined in **Scheme 1**. In our preliminary investigations of tamoxifen and its derivatives using the three-component coupling reaction [16,17,18,19,20,21,22], the obtained 3,4,4-triaryl-1-butene **A** was found to be the common skeleton of SERMs. It is anticipated that **A** would also be easily converted into a key intermediate as a precursor of **1** and **2**. The terminal olefin part in **A** might be transformed into an iodonium ion intermediate, which could react with one of the two aromatic rings at the C-1 position by electrophilic aromatic substitution to produce a cyclized structure **B**. 

After the elimination of hydrogen iodide, the generated double-bond could be migrated with a base to afford dihydronaphthalene structure **C**, which corresponds to the main frames of **1** and **2**. The successive installation of an appropriate side-chain into **C** might produce **2**. Since it is already known that **2** could be converted to **1** by hydrogenation of the double-bond as shown in the above instances, establishment of a method for the preparation of **2** would be equivalent to completion of the formal total synthesis of **1**. According to this synthetic plan, we started to develop a new method for the preparation of **1** and **2** through the three-component coupling reaction as part of our continuous efforts applying this novel protocol to produce new types of SERMs. 

### 2.1. Diversity Oriented Synthesis of the Common Frameworks of Lasofoxifene (***1***), Nafoxidine (***2***), inv-Lasofoxifene (***3***), and inv-Nafoxidine (***4***) Using the Lewis acid-Mediated Three-Component Coupling Reaction

**Scheme 2:**
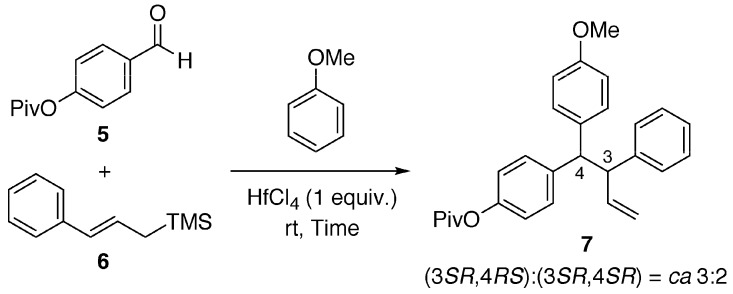
The three-component coupling reaction among 4-pivaloyloxybenzaldehyde (**5**), cinnamyltrimethylsilane (**6**), and anisole in the presence of HfCl_4_.

We first tried to optimize the reaction conditions of the three-component coupling process among4-pivaloyloxybenzaldehyde (**5**), cinnamyltrimethylsilane (**6**), and anisole (**Scheme 2**). These results are summarized in **Table 1**. Anisole was used as a solvent in this reaction, therefore, the determination of the best concentrations of substrates **5** and **6** to anisole was crucially important to attain the satisfactory yield of the desired 3,4,4-triaryl-1-butene **7**. Although the targeted molecule **7** was produced in medium yield (43%) when the reaction was carried out at a low concentration (0.1 M) in anisole including 1 molar amount of **5** and 1.2 molar amounts of **6** as shown in Entry 1, the yield of **7** increased to 63% by the reaction of 1 molar amount of **5** and 2 molar amounts of **6** at the same concentration of **5** in anisole (Entry 2). As shown in Entries 3-5, better results were obtained under the higher concentrations of **5** in anisole (0.3-0.4 M) and the coupling product **7** was obtained in good yield (72-79%) using a two-fold amount of **6** to **5**. On the other hand, the yield of the three-component coupling product **7** was lowered by rising the concentration of substrate **5** in anisole (Entries 6-8), because the amount of anisole as a second nucleophile in this reaction was inversely proportional to the concentration of **5** in anisole. 

All of the coupling reactions listed in **Table 1** afforded *ca* a 3:2 mixture of the *anti*-(3*SR*,4*RS*)- and *syn*-(3*RS*,4*RS*)-diastereomers of the 3,4,4-triaryl-1-butene. The ratio of the diastereomers was determined by 1H-NMR and HPLC analyses. Because these compounds could not be separated at this stage, a diastereomeric mixture of the coupling products was used for the next carbocyclization step without further purification. It was anticipated that both compounds might be converted into the corresponding valuable dihydronaphthalene derivatives by the double-bond migration in the later step. 

**Scheme 3:**
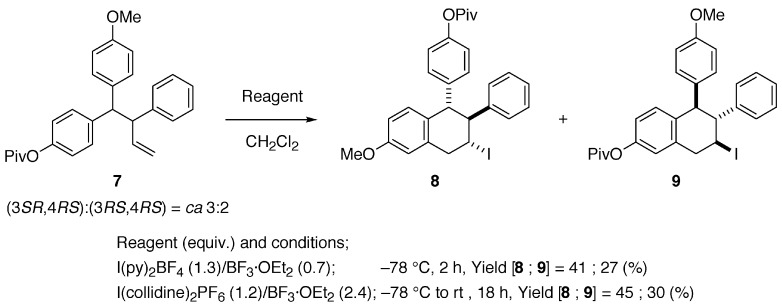
Iodine-induced cationic carbocyclization of the coupling product **7**.

Secondly, several methods for the electrophilic cyclization to form the fused compounds **8** and **9** from 3,4,4-triaryl-1-butene **7** were examined (**Scheme 3**). No transformation occurred when the BF_3_·OEt_2_-mediated reaction was first applied for the ring closure reaction of **7** according to the literature method [37,38], and the bromine-mediate carbocyclization procedure using NBS/BF_3_·OEt_2_ at −78 °C resulted in a rather complicated reaction mixture. We next tried the iodine-induced cationic cyclization of **7** using bis(pyridine)iodonium(I) tetrafluoroborate (I(py)_2_BF_4_), which was developed by Barluenga [39,40] and Casey [41]. Fortunately, the desired cyclization smoothly took place in the presence of stoichiometric amounts of I(py)_2_BF_4_/BF_3_·OEt_2_, and two bicyclic products **8** and **9** were predominantly obtained in 41% and 27% yields, respectively. We further applied bis(2,4,6-trimethylpyridine)iodonium(Ι) hexafluorophosphate (I(collidine)_2_PF_6_), which was used as electrophilic halogenation reagent by Rousseau [42], for the cyclization of **7** and the reaction also smoothly proceeded to produce **8** and **9** in 45 and 30%, respectively. It is noteworthy that both cyclized compounds **8** and **9** have all *trans* configurations and the reaction exclusively afforded these positional isomers **8** and **9**. 

**Figure 3:**
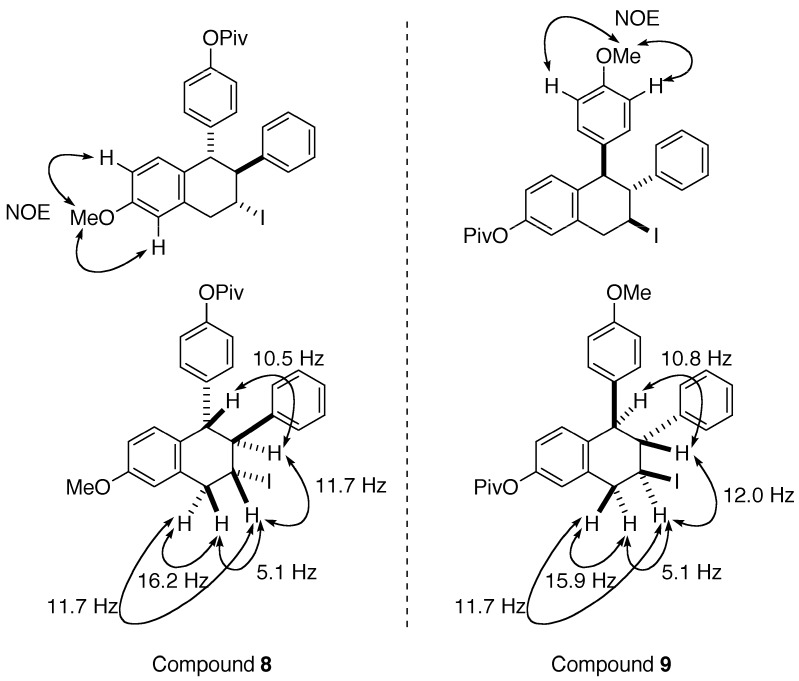
Determination of the positions of methoxy and pivaloyloxy groups in **8** and **9** and the stereochemistry of **8** and **9**.

The primary structures of the two cyclized compounds **8** and **9** listed above were confirmed by the observation of the enhanced NOEs of these products as illustrated in **Figure 3** and the positions of the methoxy and pivaloyloxy groups on the aromatic rings have been unambiguously determined. Furthermore, relative stereochemistries of these positional isomers were definitely deduced on the basis of the coupling constants of the 1H-NMR spectroscopy. 

**Scheme 4:**
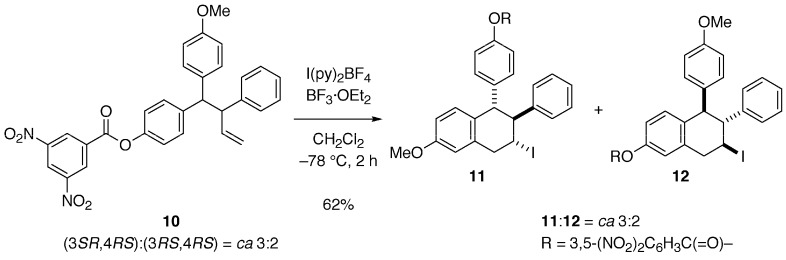
Iodine-induced cationic carbocyclization of the coupling product **10**.

To assess the electronic effect of the aromatic rings in the iodonium cation-mediated cyclization to form the positional isomers **8** and **9**, the three-component coupling product **10** having a strong electron withdrawing group at the *para*-position in one of aromatic rings was prepared and subjected to the iodine-induced cationic cyclization using I(py)_2_BF_4_ (**Scheme 4**). 

Interestingly, the carbocyclization smoothly proceeded to afford the cyclized compounds (**11** and **12**) in 62% yield (**11**:**12** = *ca* 3:2) with the same chemoselectivity, which was observed in the cyclization of **7** to produce **8** and **9** (**8**:**9** = *ca* 3:2). Therefore, it was proved that the selectivity of the cyclization of **7** to form the positional isomers **8** and **9** does not depend on the electronic effect of the substituents on the aromatic rings at the C-4 position in **7**.

**Scheme 5:**
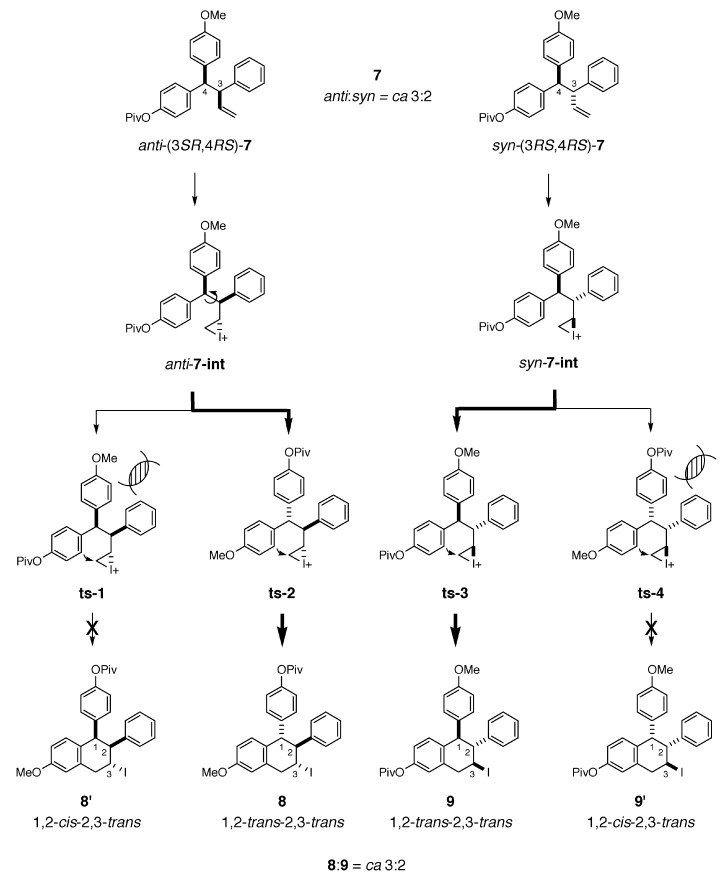
Reaction pathway to give 1,2-*trans*-2,3-*trans*-bicycles **8** and **9** from *anti*-(3*SR*,4*RS*)-**7** and *syn*-(3*RS*,4*RS*)-**7**, respectively.

Based on the above experimental results, we concluded that the ratio of **8** and **9** generated from **7** by the carbocyclization was completely reflected in that composition of the two stereoisomers, *anti*-(3*SR*,4*RS*)-**7** and *syn*-(3*RS*,4*RS*)-**7**, included in the mixture of the starting material **7**. The proposed pathways for the transformation of *anti*-(3*SR*,4*RS*)-**7** into **8** and that of *syn*-(3*RS*,4*RS*)-**7** into **9** were depicted in **Scheme 5**. The treatment of *anti*-(3*SR*,4*RS*)-**7** with iodonium cation “I+” once affords intermediate *anti*-**7-int** by the approaching of the iodonium cation from the opposite site against the aromatic ring at the C-3 position. Moreover, there exist two transition states **ts-1** and **ts-2** resulting1,2-*cis*-2,3-*trans*-bicycle **8′** and 1,2-*trans*-2,3-*trans*-bicycle **8**, respectively, by the carbocyclization of *anti*-**7-int**. Apparently, the former transition state **ts-1** has an unstable structure due to the steric repulsion between the two aromatic rings, so that another pathway to form the desired *trans*-*trans*-isomer **8** via **ts-2** would be preferable in this reaction. 

On the other hand, *syn*-**7-int** would be generated from the iodonium cation with *syn*-(3*RS*,4*RS*)-**7** by way of the similar complexation of these two components as above. There are also two transition states **ts-3** and **ts-4** which could turn into 1,2-*trans*-2,3-*trans*-bicycle **9** and 1,2-*cis*-2,3-*trans*-bicycle **9′**, respectively, by the carbocyclization of *syn*-**7-int**. The formation of an unstable transition state **ts-4** should be effectively prohibited by the existence of the neighboring two aryl groups at the C-1 and C-2 positions with *cis*-configuration; therefore, the corresponding *trans*-*trans*-isomer **9** was exclusively obtained through the transition state **ts-3** starting from *syn*-(3*RS*,4*RS*)-**7**, which was produced by the three-component coupling reaction as a minor stereoisomer. 

### 2.2. Total Synthesis of Lasofoxifene (***1***), Nafoxidine (***2***), inv-Lasofoxifene (***3***), and inv-Nafoxidine (***4***)

**Scheme 6:**
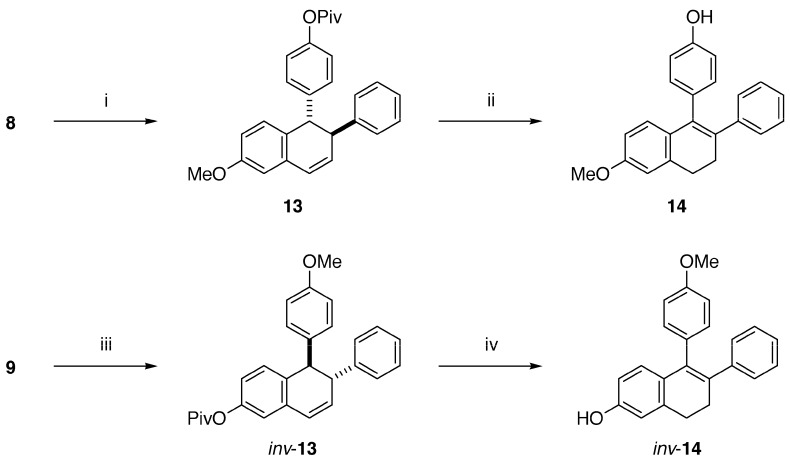
Elimination of hydrogen iodide and double-bond migration reactions.

**Scheme 7:**
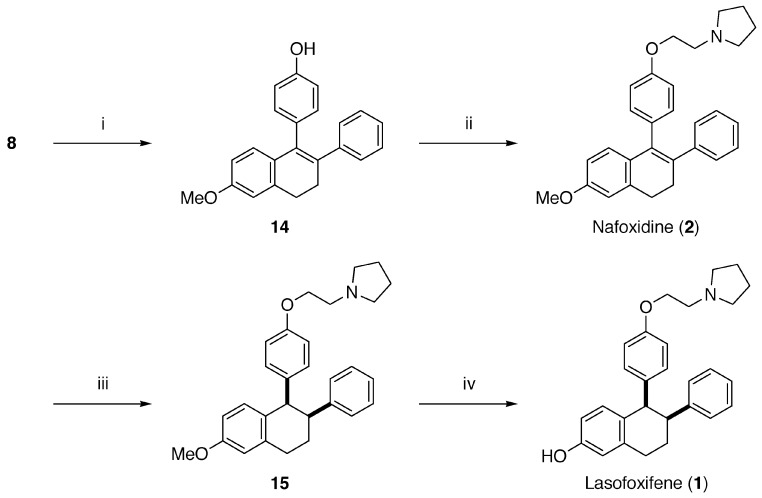
Synthesis of lasofoxifene (**1**) and nafoxidine (**2**) via the direct conversion of iodide intermediate **8** into the common precursor **14**.

**Scheme 8:**
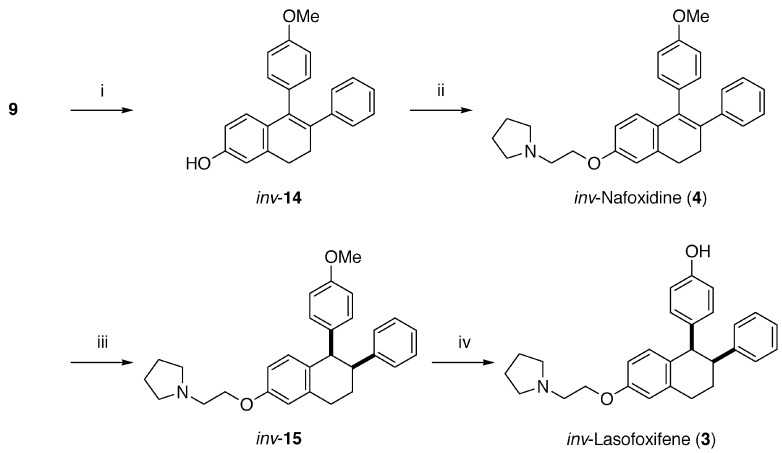
Synthesis of *inv*-lasofoxifene (**3**) and *inv*-nafoxidine (**4**) from iodine **9**.

Next, the conversion of two positional isomers **8** and **9** into the common frameworks of lasofoxifene (**1**), nafoxidine (**2**), *inv*-lasofoxifene (**3**), and *inv*-nafoxidine (**4**) was attempted as shown in **Scheme 6**, **Scheme 7** and **Scheme 8**. The dehydroiodination of **8** and **9** by treatment with DBU was preliminarily carried out to give 75% yield of the desired dihydronaphthalenes **13** and *inv*-**13** in each case (**Scheme 6**). According to our previous study, the double-bond migration of **13** and *inv*-**13** was then carried out using *t*-BuOK in DMSO and the corresponding tetra-substituted olefins **14** and *inv*-**14** having the free phenol moieties were successfully produced in good yields (70% and 69%) via the simultaneous deprotection of the pivaloyl groups. 

We further tried to explore the improved protocol to synthesize the universal precursor **14** of lasofoxifene (**1**) and nafoxidine (**2**) by the direct transformation of **8** into **14** using *t*-BuOK. During the early attempt to form **14** from **8** in the presence of *t*-BuOK in usually dried DMSO, uncharacterized over-aromatized compounds were unfortunately synthesized; however, successful conversion was finally attained using *t*-BuOK in *degassed DMSO by freeze-and-thaw cycles* as shown in **Scheme 7**. The total syntheses of nafoxidine (**2**) and lasofoxifene (**1**) were then accomplished via the successive introduction of the 2-pyrroridinoethyl moiety onto the hydroxyl group in the key intermediate **14** by the conventional method according to the report of Kapil *et al*. [43], followed by hydrogenation [33] and cleavage of the protective group in the methoxy substituent in **15** by BBr3 (**Scheme 7**). 

Finally, the positional isomers of **1** and **2**, *inv*-lasofoxifene (**3**) and *inv*-nafoxidine (**4**), were also prepared from iodine **9** in a similar manner (**Scheme 8**). It was revealed that the facile synthesis of *inv*-nafoxidine (**4**) was furnished in a 60% yield from iodine **9** via the successive olefin formation and the installation of the appropriate side-chain onto the phenol moiety of *inv*-**14**. After hydrogenating the double bond in **4** and the deprotection of methoxy group of *inv*-**15**, *inv*-lasofoxifene (**3**), one of our targeted molecules, was obtained in good yield via 2 steps from **4**. 

## 3. Experimental

### 3.1. General

All melting points are uncorrected. ^1^H- and ^13^C-NMR spectra were recorded with chloroform (in chloroform-*d*) as internal standard. Column chromatography was performed on Silica gel 60 (Merck) or Wakogel B5F. Thin layer chromatography was performed on Wakogel B5F. All reactions were carried out under argon atmosphere in dried glassware, unless otherwise noted. Dichloromethane was distilled from diphosphorus pentoxide, then calcium hydride, and dried over MS 4A, toluene and anisole were distilled from sodium, and dried over MS 4A, and DMF and DMSO were distilled from calcium hydride, and dried over MS 4A. All reagents were purchased from Tokyo Kasei Kogyo Co., Ltd (TCI), Kanto Chemical Co., Inc. or Aldrich Chemical Co., Inc., and used without further purification unless otherwise noted. Cinnamyltrimethylsilane was purchased from Junsei Chemical Co., Ltd. 

*(3SR,4RS)/(3RS,4RS)-4-(4-Methoxyphenyl)-4-([4-pivaloyloxy]phenyl)-3-phenylbut-1-ene* (**7**). 

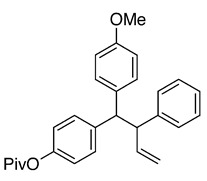



To a suspension of hafnium tetrachloride (292.6 mg, 0.9135 mmol) in anisole (1 mL) at room temperature was slowly added a mixture of cinnamyltrimethylsilane (**6**, 346.4 mg, 1.820 mmol) and 4-pivaloyloxybenzaldehyde (**5**, 188.4 mg, 0.9134 mmol) in anisole (2 mL) with cooling in a water bath to maintain the reaction mixture at room temperature. The reaction mixture was stirred for 1 h at room temperature and then saturated aqueous sodium hydrogencarbonate was added. The mixture was extracted with diethyl ether and dried over sodium sulfate. After filtration of the mixture and evaporation of the solvent, the crude product was purified by silica gel column chromatography (ethyl acetate/hexane = 1/20 to 1/9) to afford the coupling product **7** (300.6 mg, 79%, (*o*-)/(*p*-) = 2:98, (3*SR*,4*RS*)/(3*RS*,4*RS*) = *ca* 3:2) as a colorless oil; IR (neat): 2974, 1749, 1610, 1511, 1252, 1203, 1167, 1120, 1032, 913, 818, 753, 700 cm−1; ^1^H-NMR (CDCl_3_): δ 7.35–7.27 (m, 1H, Ar), 7.26–6.94 (m, 9H, Ar), 6.86–6.73 (m, 2H, Ar), 6.64–6.57 (m, 1H, Ar), 6.00–5.80 (m, 1H, 2-H), 4.97–4.80 (m, 2H, 1-H), 4.26 (d, *J* = 11.4 Hz, 1H, 3-H), 4.08 (dd, *J* = 11.4, 7.9 Hz, 1H, 4-H), 3.75 and 3.64 (s, 3H, OMe), 1.33 and 1.26 (s, 9H, CMe_3_); ^13^C-NMR (CDCl_3_): δ 177.0 and 176.8, 158.0 and 157.5, 149.3 and 148.9, 142.7 and 142.6, 141.1, 140.84 and 140.79, 135.3 and 135.1, 129.5 and 129.3, 129.1 and 129.0, 128.30 and 128.28, 128.25 and 128.20, 126.1 and 126.0, 121.2 and 120.9, 116.0 and 115.9, 113.8 and 113.5, 55.44 and 55.41, 55.1 and 55.0, 54.64 and 54.60, 39.0 and 38.9, 27.1 and 27.0; HR-MS: calcd for C_28_H_30_O_3_Na (M + Na^+^) 437.2087, found 437.2101.

*(1SR,2SR,3RS)-3-Iodo-2-phenyl-1-([4-pivaloyloxy]phenyl)-(6-methoxy-1,2,3,4-tetrahydronaphthalene* (**8**) and *(1RS,2RS,3SR)-3-Iodo-2-phenyl-1-([4-methoxy]phenyl)-(6-pivaloyloxy-1,2,3,4-tetrahydro-naphthalene)* (**9**): To a suspension of I(py)_2_BF_4_ (57.1 mg, 0.154 mmol) in CH_2_Cl_2_ (3.4 mL) at −78 °C were slowly added the coupling product 7 (56.0 mg, 0.135 mmol) in CH_2_Cl_2_ (3.4 mL) and BF_3_·OEt_2_ (150 μL, 0.118 mmol). The reaction mixture was stirred for 2 h at the same temperature and then saturated aqueous ammonium chloride was added. The mixture was extracted with diethyl ether, and the organic layer was washed with brine, dried over sodium sulfate. After filtration of the mixture and evaporation of the solvent, the crude product was purified by thin layer chromatography (toluene) to afford iodine 8 (29.9 mg, 41%) as a pale vermilion solid and its positional isomer 9 (20.0 mg, 27%) as an amorphous product, respectively. 

*(1SR,2SR,3RS)-3-Iodo-2-phenyl-1-([4-pivaloyloxy]phenyl)-(6-methoxy-1,2,3,4-tetrahydronaphthalene)* (**8**). 

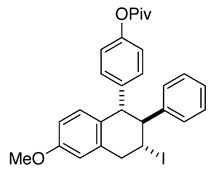



Mp. 66–68 °C; IR (KBr): 2971, 1750, 1610, 1503, 1271, 1119, 1032 cm−1; ^1^H-NMR (CDCl_3_):δ 7.16–7.07 (m, 3H, Ar), 6.84–6.77 (m, 2H, Ar), 6.75–6.63 (m, 4H, Ar), 6.54 (br s, 3H, Ar), 4.68 (ddd, *J* = 11.7, 11.7, 5.1 Hz, 1H, 3-H), 4.13 (d, *J* = 10.5 Hz, 1H, 1-H), 3.76 (dd, *J* = 16.2, 11.7 Hz, 1H,4-H*α*), 3.67 (s, 3H, OMe), 3.65 (dd, *J* = 16.2, 5.1 Hz, 1H, 4-H*β*), 3.21 (dd, *J* = 11.7, 10.5 Hz, 1H, 2-H), 1.23 (s, 9H, CMe_3_); ^13^C-NMR (CDCl_3_): δ 177.0, 157.9, 149.4, 144.4, 142.0, 137.3, 131.3, 130.8, 129.8, 128.2, 127.6, 127.0, 121.0, 113.2, 111.5, 60.0, 55.3, 53.4, 45.1, 39.0, 32.7, 27.1; HR-MS: calcd for C_28_H_29_O_3_INa (M + Na^+^) 563.1054, found 563.1055. 

*(1RS,2RS,3SR)-3-Iodo-1-([4-methoxy]phenyl)-2-phenyl-(6-pivaloyloxy-1,2,3,4-tetrahydronaphthalene)* (**9**). 

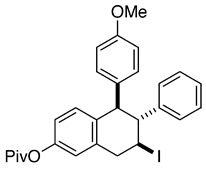



IR (neat): 2972, 1750, 1603, 1571, 1504, 1266, 1202, 1119, 1032 cm−1; ^1^H-NMR (CDCl^3^): δ 7.23–7.16 (m, 3H, Ar), 6.92–6.86 (m, 2H, Ar), 6.83–6.79 (br s, 1H, Ar), 6.76–6.71 (m, 2H, Ar), 6.69–6.62 (m, 4H, Ar), 4.77 (ddd, *J* = 12.0, 11.7, 5.1 Hz, 1H, 3-H), 4.19 (d, *J* = 10.2 Hz, 1H, 1-H), 3.87 (dd, *J* = 15.9, 11.7 Hz, 1H, 4-H*α*), 3.75 (dd, *J* = 15.9, 5.1 Hz, 1H, 4-H*β*), 3.72 (s, 3H, OMe), 3.32 (dd, *J* = 12.0, 10.2 Hz, 1H, 2-H), 1.35 (s, 9H, CMe_3_); ^13^C-NMR (CDCl_3_): δ 177.2, 158.0, 149.2, 144.5, 137.3, 136.6, 136.3, 131.2, 129.9, 128.1, 127.6, 126.9, 119.9, 119.8, 113.4, 59.9, 55.1, 53.3, 44.8, 39.0, 32.2, 27.1; HR-MS: calcd for C_28_H_29_O_3_INa (M + Na^+^) 563.1054, found 563.1080. 

*(1SR,2SR)-2-Phenyl-1-([4-pivaloyloxy]phenyl)-(6-methoxy-1,2-dihydronaphtalene)* (**13**). 

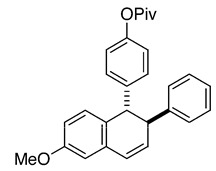



To a solution of iodine **8** (39.4 mg, 0.0729 mmol) in toluene (1.5 mL) at room temperature was added DBU (35.0 μL, 0.234 mmol). The reaction mixture was stirred for 15 min at 95 °C and then saturated aqueous ammonium chloride was added at 0 °C. The mixture was extracted with diethyl ether, and the organic layer was washed with brine, dried over sodium sulfate. After filtration of the mixture and evaporation of the solvent, the crude product was purified by thin layer chromatography (benzene/hexane = 10:1) to afford dihydronaphtalene **13** (22.7 mg, 75%) as a colorless oil; IR (neat): 2972, 1750, 1610, 1511, 1243, 1122, 1031 cm−1; ^1^H-NMR (CDCl_3_): δ 7.24–7.06 (m, 7H, Ar), 6.95–6.59 (m, 6H, 4-H, Ar), 5.98 (dd, *J* = 9.6, 4.2 Hz, 1H, 3-H), 4.17 (d, *J* = 7.5 Hz, 1H, 1-H), 3.82 (ddd, *J* = 7.5, 4.2, 2.1 Hz, 1H, 2-H), 3.80 (s, 3H, OMe), 1.34 (s, 9H, CMe_3_); ^13^C-NMR (CDCl_3_): δ 177.1, 158.6, 149.4, 143.7, 142.0, 134.4, 130.9, 129.8, 129.3, 128.4, 128.2, 127.9, 127.8, 126.5, 121.2, 112.7, 111.9, 55.3, 51.2, 49.6, 39.0, 27.1; HR-MS: calcd for C_28_H_28_O_3_Na (M + Na^+^) 435.1931, found 435.1946. 

*(1RS,2RS)-1-([4-Methoxy]phenyl)-2-phenyl-(6-pivaloyloxy-1,2-dihydronaphtalene)* (*inv*-**13**). 

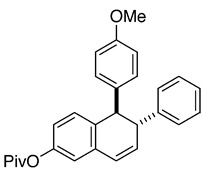



To a solution of iodine **9** (28.1 mg, 0.0520 mmol) in toluene (1 mL) at room temperature was added DBU (25.0 μL, 0.167 mmol). The reaction mixture was stirred for 15 min at 95 °C and then saturated aqueous ammonium chloride was added at 0 °C. The mixture was extracted with diethyl ether, and the organic layer was washed with brine, dried over sodium sulfate. After filtration of the mixture and evaporation of the solvent, the crude product was purified by thin layer chromatography (benzene/hexane = 10:1) to afford dihydronaphtalene *inv*-**13** (16.0 mg, 75%) as a colorless oil; IR (neat): 3029, 2973, 2933, 1750, 1510, 1491, 1246, 1148, 1121, 1032, 703 cm^−1^; ^1^H-NMR (CDCl_3_):δ 7.24–6.97 (m, 7H, Ar), 6.89 (d, *J* = 2.1 Hz, 1H, Ar), 6.83–6.72 (m, 4H, Ar), 6.62 (dd, *J* = 9.6, 1.8 Hz, 1H, 4-H), 6.01 (dd, *J* = 9.6, 4.2 Hz, 1H, 3-H), 4.16 (d, *J* = 7.8 Hz, 1H, 1-H), 3.84 (ddd, *J* = 7.8, 4.2, 1.8 Hz, 1H, 2-H), 3.76 (s, 3H, OMe), 1.36 (s, 9H, CMe_3_); ^13^C-NMR (CDCl_3_): δ 177.2, 158.1, 149.9, 143.7, 136.2, 134.5, 133.9, 131.4, 129.7, 129.4, 128.4, 127.9, 127.2, 126.5, 120.2, 119.0, 113.7, 55.2, 51.1, 49.5, 39.1, 27.1; HR-MS: calcd for C_28_H_28_O_3_Na (M + Na^+^) 435.1931, found 435.1940. 

*1-([4-Hydroxy]phenyl)-2-phenyl-(6-methoxy-3,4-dihydronaphtalene)* (**14**).

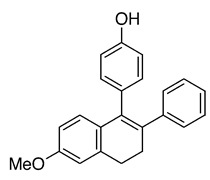



(Method A; Stepwise procedure from **13**)

To a solution of potassium *tert*-butoxide (40.3 mg, 0.359 mmol) in DMSO (0.6 mL) at room temperature was added dihydronaphtalene **13** (29.3 mg, 0.071 mmol) in DMSO (0.8 mL). The reaction mixture was stirred for 21 h at room temperature and then saturated aqueous ammonium chloride was added at 0 °C. The mixture was extracted with diethyl ether, and the organic layer was washed with brine, dried over sodium sulfate. After filtration of the mixture and evaporation of the solvent, the crude product was purified by thin layer chromatography (toluene/ethyl acetate = 10:1) to afford the common intermediate **14** (16.0 mg, 70%) as a pale yellow oil; ^1^H-NMR (CDCl_3_): δ 7.19–6.99 (m, 5H, Ar), 6.94–6.88 (m, 2H, Ar), 6.82–6.66 (m, 4H, Ar), 6.60 (dd, *J* = 8.4, 3.0 Hz, 1H, Ar), 5.48 (br s, 1H, OH), 3.81 (s, 3H, OMe), 2.99–2.87 (m, 2H, 4-H), 2.83–2.72 (m, 2H, 3-H). Above prepared **14** was instantly used in the following reaction as soon as possible. 

(Method B; One-step procedure from **8**)

To iodine **8** (74.4 mg, 0.138 mmol) at room temperature was added a solution of potassium *tert*-butoxide in *degassed* DMSO (0.5 M, 1.10 mL, 0.550 mmol). The reaction mixture was stirred for 1 h at 90 °C and then saturated aqueous ammonium chloride was added at 0 °C. The mixture was extracted with diethyl ether/hexane = 1:1, and the organic layer was dried over sodium sulfate. After filtration of the mixture and evaporation of the solvent, the crude product was purified by silica gel column chromatography (hexane/ethyl acetate = 4:1) to afford the common intermediate **14** (41.1 mg, 89%) as a pale yellow oil. Above prepared **14** was instantly used in the following reaction as soon as possible. 

*1-([4-Methoxy]phenyl)-2-phenyl-(6-hydroxy-3,4-dihydronaphtalene)* (*inv*-**14**).

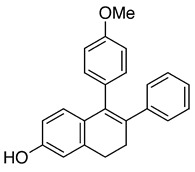



(Method A; Stepwise procedure from *inv*-**13**)

To a solution of potassium *tert*-butoxide (31.7 mg, 0.282 mmol) in DMSO (0.32 mL) at room temperature was added dihydronaphtalene *inv*-**13** (14.9 mg, 0.0361 mmol) in DMSO (0.40 mL). The reaction mixture was stirred for 3 h at 45 °C and then saturated aqueous ammonium chloride was added at 0 °C. The mixture was extracted with diethyl ether, and the organic layer was dried over sodium sulfate. After filtration of the mixture and evaporation of the solvent, the crude product was purified by thin layer chromatography (toluene/ethyl acetate = 10:1) to afford the common intermediate *inv*-**14** (8.2 mg, 69%) as a pale yellow oil; ^1^H-NMR (CDCl_3_): δ 7.12–6.81 (m, 7H, Ar), 6.70–6.53 (m, 4H, Ar), 6.40 (dd, *J* = 8.4, 2.4 Hz, 1H, Ar), 5.10 (br s, 1H, OH), 3.67 (s, 3H, OMe), 2.82–2.76 (m, 2H, 4-H), 2.71–2.63 (m, 2H, 3-H). Above prepared *inv*-**14** was instantly used in the following reaction as soon as possible. 

(Method B; One-step procedure from **9**)

To iodine **9** (48.3 mg, 0.0894 mmol) at room temperature was added a solution of potassium *tert*-butoxide in *degassed* DMSO (0.5 M, 0.715 mL, 0.358 mmol). The reaction mixture was stirred for 1 h at 90 °C and then saturated aqueous ammonium chloride was added at 0 °C. The mixture was extracted with diethyl ether/hexane = 1:1, and the organic layer was washed with brine, dried over sodium sulfate. After filtration of the mixture and evaporation of the solvent, the crude intermediate *inv*-**14** was used for the next reaction to provide **4** without further purification. 

*Nafoxidine* (**2**). 

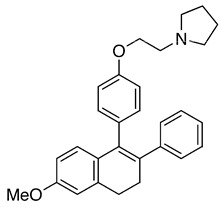



To a solution of alcohol **14** (31.7 mg, 0.0965 mmol) in DMF (1 mL) at room temperature was gradually added sodium hydride (60%, 13.4 mg, 0.335 mmol). After the reaction mixture had been stirred for 20 min at the same temperature, 1-(2-chloroethyl)pyrrolidine hydrochloride (33.4 mg, 0.196 mmol) was added to the reaction mixture. The reaction mixture was stirred for 11 h at 50 °C and then saturated aqueous sodium hydrogencarbonate was added at 0 °C. The mixture was extracted with ethyl acetate, and dried over sodium sulfate. After filtration of the mixture and evaporation of the solvent, the crude product was purified by thin layer chromatography (hexane/ethyl acetate/35% NH_3_ = 7:3:1) to afford nafoxidine (**2**) (33.5 mg, 82%) as a pale yellow oil; IR (neat): 3031, 2936, 1669, 1606, 1568, 1508, 1241, 1174, 1037 cm^−1^; ^1^H-NMR (CDCl_3_): δ 7.14–6.92 (m, 7H, Ar), 6.80–6.69 (m, 4H, Ar), 6.59 (dd, *J* = 9.0, 2.5 Hz, 1H, Ar), 4.18 (t, *J* = 6.0 Hz, 2H, OCH_2_), 3.80 (s, 3H, OMe), 3.18–3.01 (m, 2H, NCH_2_), 2.94–2.72 (m, 8H, 3-H, 4-H, pyrrolidinyl 2-H), 1.94–1.88 (m, 4H, pyrrolidinyl 3-H);^13^C-NMR (CDCl_3_): δ 158.4, 157.0, 143.2, 137.7, 134.6, 134.3, 132.2, 130.4, 128.2, 127.60, 127.55, 127.4, 125.6, 114.0, 113.2, 110.8, 65.9, 55.3, 54.8, 54.6, 30.7, 29.0, 23.4; HR-MS: calcd for C_29_H_32_O_2_N (M + H^+^) 426.2428, found 426.2430. 

inv-Nafoxidine (**4**). 

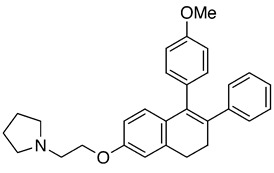



To a solution of the crude *inv*-**14** derived from iodine **9** in DMF (1 mL) at room temperature was gradually added sodium hydride (60%, 10.7 mg, 0.268 mmol). After the reaction mixture had been stirred for 20 min at the same temperature, 1-(2-chloroethyl)pyrrolidine hydrochloride (27.4 mg,0.161 mmol) was added to the reaction mixture. The reaction mixture was stirred at 50 °C for 11 h and then saturated aqueous sodium hydrogen carbonate was added at 0 °C. The mixture was extracted with ethyl acetate, and dried over sodium sulfate. After filtration of the mixture and evaporation of the solvent, the crude product was purified by thin layer chromatography (CHCl_3_/MeOH = 9:1) to afford *inv*-nafoxidine (**4**) (22.7 mg, 60% from **9**) as a pale yellow oil; IR (neat): 2963, 2784, 1606, 1511, 1243, 1036, 831, 758, 700 cm^−1^; ^1^H-NMR (CDCl_3_): δ 7.17–6.93 (m, 7H, Ar), 6.82–6.69 (m, 4H, Ar), 6.61 (dd, *J* = 8.4, 2.7 Hz, 1H, Ar), 4.11 (t, *J* = 6.0 Hz, 2H, OCH_2_), 3.78 (s, 3H, OMe), 3.00–2.85 (m, 4H, 4-H, NCH_2_), 2.82–2.73 (m, 2H, 3-H), 2.72–2.56 (m, 4H, pyrrolidinyl 2-H), 1.87–1.75 (m, 4H, pyrrolidinyl 3-H); ^13^C-NMR (CDCl_3_): δ 158.0, 157.6, 143.3, 137.6, 134.7, 134.2, 132.1, 132.0, 130.5, 128.2, 127.5, 127.4, 125.6, 113.8, 113.3, 111.4, 67.0, 55.08, 55.06, 54.7, 30.7, 28.9, 23.4; HR-MS: calcd for C_29_H_32_O_2_N (M + H^+^) 426.2428, found 426.2438. 

*(1RS,2SR)-1-(4-[2-Pyrrolidinoethoxy]phenyl)-2-phenyl-(6-methoxy-1,2,3,4-tetrahydronaphtalene)* (**15**). 

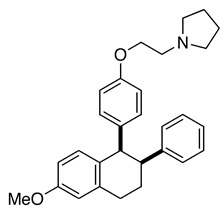



A 10 mL autoclave was charged with nafoxidine (**2**, 36.0 mg, 0.0846 mmol), 20% Pd(OH)2/C (36.0 mg, 0.0513 mmol), and EtOH (3.0 mL). The vessel was sealed and then the whole mixture was stirred for 22 h at 50 °C under hydrogen atmosphere (2.5 atm). After cooling to room temperature, the reactant was filtered through a short pad of Celite with EtOAc at room temperature under ambient atmosphere. The filtrate was concentrated and purified by silica gel chromatography (hexane/ethyl acetate/35% NH_3_ = 7:3:1) to afford **15** (25.2 mg, 70%) as a colorless oil; IR (neat): 2932, 1608, 1506, 1460, 1240, 1178, 1155, 1038, 823 cm^−1^; ^1^H-NMR (CDCl_3_): δ 7.21–7.12 (m, 3H, Ar), 6.90–6.64 (m, 5H, Ar), 6.59–6.52 (m, 2H, Ar), 6.36–6.27 (m, 2H, Ar), 4.24 (d, *J* = 5.1 Hz, 1H, 1-H), 4.00 (t, *J* = 6.0 Hz, 1H, OCH_2_), 3.82 (s, 3H, OMe), 3.36 (ddd, *J* = 12.9, 5.1, 2.1 Hz, 1H, 2-H), 3.15–3.06 (m, 2H, 4-H), 2.85 (t, *J* = 6.0 Hz, 2H, NCH_2_), 2.70–2.56 (m, 4H, pyrrolidinyl 2-H), 2.26–2.11 (m, 1H, 3-H), 1.89–1.76 (m, 5H, 3-H, pyrrolidinyl 3-H); ^13^C-NMR (CDCl_3_): δ 157.8, 156.8, 144.3, 137.7, 134.7, 132.2, 131.5, 131.3, 128.1, 127.7, 125.9, 112.94, 112.91, 112.5, 66.5, 55.2, 55.0, 54.6, 50.2, 45.4, 30.1, 23.4, 21.9; HR-MS: calcd for C_29_H_34_O_2_N (M + H^+^) 428.2584, found 428.2586. 

*(1RS,2SR)-1-(4-[Methoxy]phenyl)-2-phenyl-(6-[2-pyrrolidinoethoxy]-1,2,3,4-tetrahydronaphtalene)* (*inv*-**15**). 

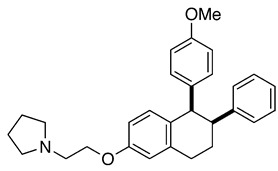



A 10 mL autoclave was charged with *inv*-nafoxidine (**4**, 14.9 mg, 0.0350 mmol), 20% Pd(OH)2/C (15.0 mg, 0.0214 mmol), and EtOH (1.5 mL). The vessel was sealed and then the whole mixture was stirred for 24 h at 50 °C under hydrogen atmosphere (2.5 atm). After cooling to room temperature, the reactant was filtered through a short pad of Celite with EtOAc at room temperature under ambient atmosphere. The filtrate was concentrated and purified by silica gel chromatography (hexane/ethyl acetate/35% NH_3_ = 7:3:1) to afford *inv*-**15** (11.8 mg, 79%) as a colorless oil; IR (neat): 2931, 1608, 1505, 1459, 1242, 1037, 823, 755, 700 cm^−1^; ^1^H-NMR (CDCl_3_): δ 7.20–7.13 (m, 3H, Ar), 6.86 (d,*J* = 8.0 Hz, 1H, Ar), 6.83–6.77 (m, 3H, Ar), 6.70 (dd, *J* = 8.0, 2.5 Hz, 1H, Ar), 6.57–6.52 (m, 2H, Ar), 6.36–6.29 (m, 2H, Ar), 4.24 (d, *J* = 5.0 Hz, 1H, 1-H), 4.16–4.08 (m, 2H, OCH_2_), 3.70 (s, 3H, OMe), 3.36 (ddd, *J* = 11.3, 5.0, 2.0 Hz, 1H, 2-H), 3.10–3.01 (m, 2H, 4-H), 2.91 (t, *J* = 6.0 Hz, 2H, NCH_2_), 2.70–2.61 (m, 4H, pyrrolidinyl 2-H), 2.26–2.11 (m, 1H, 3-H), 1.87–1.76 (m, 5H, 3-H, pyrrolidinyl 3-H); ^13^C-NMR (CDCl_3_): δ 157.5, 157.1, 144.3, 137.7, 134.6, 132.3, 131.4, 131.3, 128.1, 127.7, 125.9, 113.7, 113.1, 112.2, 66.9, 55.1, 55.0, 54.7, 50.1, 45.4, 30.1, 23.5, 21.9; HR-MS: calcd for C_23_H_21_O_2_Na [M – (C_4_H_8_N + CH_2_ = CH_2_) + Na^+^] 352.1434, found 352.1427. 

*Lasofoxifene* (**1**). 

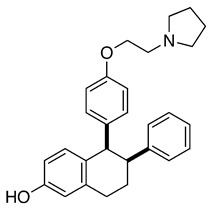



To a solution of methyl ether **15** (10.1 mg, 0.0236 mmol) in CH2Cl2 (0.6 mL) at –78 °C was slowly added BBr3 in CH2Cl2 (1.0 M, 0.120 mL, 0.120 mmol). The reaction mixture was stirred for 1 h at–23 °C and 2 h at 0 °C and then saturated aqueous sodium hydrogencarbonate was added at the same temperature. The mixture was extracted with ethyl acetate, and the organic layer was washed with brine, dried over sodium sulfate. After filtration of the mixture and evaporation of the solvent, the crude product was purified by thin layer chromatography (hexane/ethyl acetate/35% NH_3_ = 3:6:1) to afford lasofoxifene (**1**, 7.4 mg, 76%) as a colorless oil; IR (neat): 3471, 2928, 2873, 1668, 1506, 1455, 1177, 1035, 823, 757 cm^−1^; ^1^H-NMR (CDCl_3_): δ 7.11–7.00 (m, 3H, Ar), 6.75–6.63 (m, 3H, Ar), 6.53 (d, *J* = 2.4 Hz, 1H, Ar), 6.46 (dd, *J* = 8.1, 2.4 Hz, 1H, Ar), 6.27 (d, *J* = 8.7 Hz, 2H, Ar), 6.16 (d, *J* = 8.7 Hz, 2H, Ar), 5.20 (br s, 1H, OH), 4.10 (d, *J* = 4.8 Hz, 1H, 1-H), 3.99–3.83 (m, 2H, OCH_2_), 3.24 (ddd, *J* = 11.4, 4.8, 2.0 Hz, 1H, 2-H), 3.00–2.60 (m, 8H, 4-H, NCH_2_, pyrrolidinyl 2-H), 2.07–1.95 (m, 1H, 3-H), 1.87–1.76 (m, 5H, 3-H, pyrrolidinyl 3-H); ^13^C-NMR (CDCl_3_): δ 156.6, 155.0, 144.5, 137.6, 134.9, 131.5, 131.2, 128.3, 128.2, 127.7, 125.9, 114.9, 114.0, 112.7, 65.6, 55.1, 54.4, 50.2, 45.4, 29.9, 23.2, 21.9; HR-MS: calcd for C_28_H_32_O_2_N (M + H^+^) 414.2428, found 414.2436. 

*inv-Lasofoxifene* (**3**). 

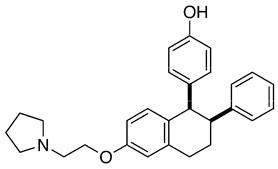



To a solution of methyl ether *inv*-**15** (11.8 mg, 0.0276 mmol) in CH2Cl2 (0.7 mL) at −78 °C was slowly added BBr3 in CH2Cl2 (1.0 M, 0.140 mL, 0.140 mmol). The reaction mixture was stirred for 1 h at –78 °C and 4 h at 0 °C and then saturated aqueous sodium hydrogencarbonate was added at the same temperature. The mixture was extracted with ethyl acetate, and the organic layer was washed with brine, dried over sodium sulfate. After filtration of the mixture and evaporation of the solvent, the crude product was purified by thin layer chromatography (hexane/ethyl acetate/35% NH_3_ = 3:6:1) to afford *inv*-lasofoxifene (**3**, 7.9 mg, 69%) as a colorless oil; IR (neat): 3362, 2923, 1776, 1713, 1663, 1609, 1241, 1119, 1051, 991, 756 cm^−1^; ^1^H-NMR (CDCl_3_): δ 7.20–7.11 (m, 3H, Ar), 6.85–6.77 (m, 3H, Ar), 6.73 (d, *J* = 2.5 Hz, 1H Ar), 6.60–6.55 (m, 1H, Ar), 6.47–6.43 (m, 2H, Ar), 6.26–6.23 (m, 2H, Ar), 5.42 (br s, 1H, OH), 4.21 (d, *J* = 5.0 Hz, 1H, 1-H), 4.16–4.06 (m, 2H, OCH_2_), 3.37–3.30 (m, 1H, 2-H), 3.09–2.90 (m, 4H, 4-H, NCH_2_), 2.61–2.54 (m, 4H, pyrrolidinyl 2-H), 2.22–2.12 (m, 2H, 3-H), 1.89–1.77 (m, 4H, pyrrolidinyl 3-H); ^13^C-NMR (CDCl_3_): δ 157.0, 154.0, 144.4, 137.6, 134.3, 132.4, 131.5, 131.4, 128.2, 127.7, 125.9, 114.0, 113.6, 113.1, 66.5, 55.1, 54.6, 50.2, 45.4, 30.1, 23.4, 21.9; HR-MS: calcd for C_28_H_32_O_2_N (M + H^+^) 414.2428, found 414.2432. 

## 4. Conclusions

Thus, we developed a new method to produce lasofoxifene (**1**), nafoxidine (**2**) and their positional isomers *inv*-lasofoxifene (**3**) and *inv*-nafoxidine (**4**) using the three-component coupling reaction among 4-pivaloyloxybenzaldehyde (**5**), cinnamyltrimethylsilane (**6**), and anisole in the presence of HfCl_4_. The intermediate 3,4,4-triaryl-1-butene **7** was effectively transformed into **2** and **4**, which were precursors of **1** and **3**, respectively, via the successive three-step transformations; namely, electrophilic carbocyclization, sequential double-bond formation/migration, and side-chain installation. This synthetic strategy seems to serve as a novel and practical pathway to prepare not only the nafoxidine and lasofoxifene derivatives, but also new entries as SERM-type drugs including estrogen-dependent breast cancer, leukemia, and osteoporosis agents.

## Figures and Tables

*Reagents and conditions*: (i) DBU (3.2 equiv.), toluene, 95 °C, 15 min, 75%; (ii) *t*-BuOK (5.0 equiv.), DMSO, rt, 21 h, 70%; (iii) DBU (3.2 equiv.), toluene, 95 °C, 15 min, 75%; (iv) *t*-BuOK (7.8 equiv.), DMSO, 45 °C, 3 h, 69%.

*Reagents and conditions*: (i) *t*-BuOK (4.0 equiv.), *degassed* DMSO, 90 °C, 1 h, 89%; (ii) Cl(CH_2_)_2_N(CH_2_)_2_·HCl (2.0 equiv.), NaH (3.5 equiv.), DMF, 50 °C, 11 h, 82%; (iii) H_2_ (2.5 atm), Pd(OH)_2_/C, EtOH, 50 °C, 22 h, 70%; (iv) BBr_3_ (5.0 equiv.), CH_2_Cl_2_, −23 to 0 °C, 3 h, 76%.

*Reagents and conditions*: (i) *t*-BuOK (4.0 equiv.), *degassed* DMSO, 90 °C, 1 h; (ii) Cl(CH_2_)_2_N(CH_2_)_2_·HCl (1.8 equiv.), NaH (3.0 equiv.), DMF, 50 °C, 11 h, 60% (2 steps); (iii) H2 (2.5 atm), Pd(OH)_2_/C, EtOH, 50 °C, 24 h, 79%; (iv) BBr_3_ (5.0 equiv.), CH_2_Cl_2_, −78 to 0 °C, 5 h, 69%.

**Table 1:** Synthesis of 3,4,4-triaryl-1-butene **7** by the three-component coupling reaction among 4-pivaloyloxybenzaldehyde (**5**), cinnamyltrimethylsilane (**6**), and anisole in the presence of HfCl_4_.*^a^*

Entry	**5** / **6** (equiv.)	Conc. (mol/L)	Time (h)	Yield of **7** (%)*b*
1	1/1.2	0.1	20	43
2	1/2	0.1	20	63
3	1/2	0.3	1	79
4	1/2	0.3	2	78
5	1/2	0.4	2	72
6	1/2	0.6	20	67
7	1/2	0.7	20	56
8	1/2	0.8	20	54

*a* All new compounds were characterized by ^1^H NMR, ^13^C NMR spectroscopy and mass spectroscopy. *b* Isolated yields after purification by silica gel chromatography.

## Supplementary Materials

Spectroscopic data of synthetic intermediates and products is available free of charge at http://www.mdpi.com/1420-3049/15/10/6673/s1.
