# Supplementary material for: Synthesis of Lasofoxifene, Nafoxidine and Their Positional Isomers via the Novel Three-Component Coupling Reaction
Source: Molecules. 2010 Sep 28;15(10):6773–94. doi: 10.3390/molecules15106773 (PMC6259163; doi:10.3390/molecules15106773)
Supplement: Supplementary file 1 [file molecules-15-06773-s001.pdf]

*Molecules* **2010**, *15*

OPEN ACCESS

***molecules***

**ISSN 1420-3049**

www.mdpi.com/journal/molecules

*Supporting Information*

# Synthesis of Lasofoxifene, Nafoxidine and Their Positional Isomers via the Novel Three-Component Coupling Reaction

**Kenya Nakata, Yoshiyuki Sano and Isamu Shiina ***

Department of Applied Chemistry, Faculty of Science, Tokyo University of Science, 1-3 Kagurazaka, Shinjuku-ku, Tokyo 162-8601, Japan

* Author to whom correspondence should be addressed; E-Mail: shiina@rs.kagu.tus.ac.jp; Fax: +81-3-3260-5609.

*Received: 21 August 2010; in revised form: 13 September 2010 / Accepted: 20 September 2010/ Published: 28 September 2010*

---

## Supplementary Material

S2-25 $^{1}H$ and $^{13}C$ NMR Spectroscopic Data of Compounds

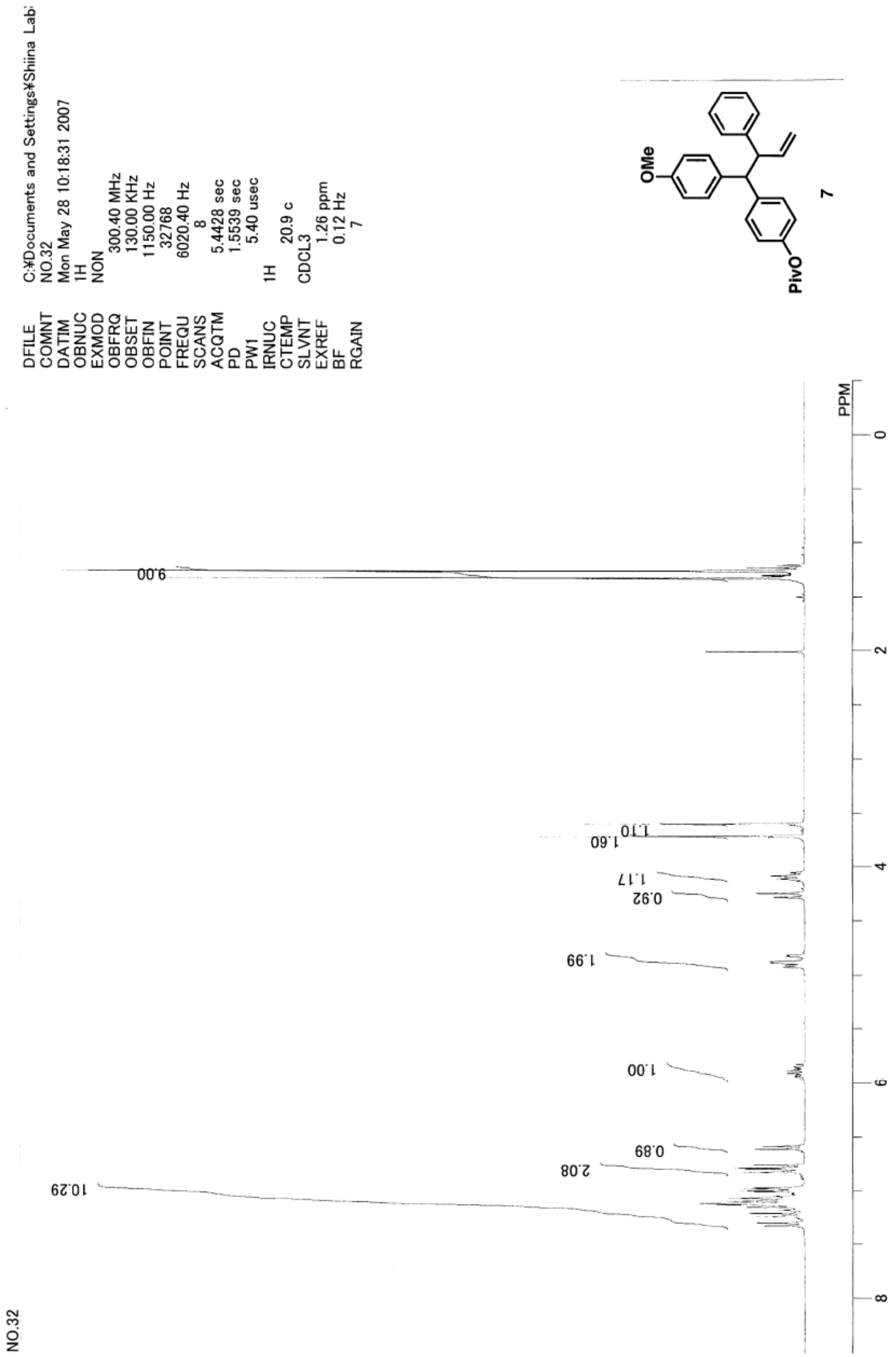
DFILE C:¥Documents and Settings¥Shiina Lab¥
COMNT NO.32
DATIM Mon May 28 10:18:31 2007
OBNUC 1H
EXMOD NON
OBFRQ 300.40 MHz
OBSET 130.00 KHz
OBFIN 1150.00 Hz
POINT 32768
FREQU 6020.40 Hz
SCANS 8
ACQTM 5.4428 sec
PD 1.5539 sec
PW1 5.40 usec
IRNUC 1H
CTEMP 20.9 c
SLVNT CDCL3
EXREF 1.26 ppm
BF 0.12 Hz
RGAIN 7
OMe
PivO
7
NO.32
10.29
2.08
0.89
1.00
1.99
0.92
1.17
1.60
1.10
9.00
PPM
0
2
4
6
8

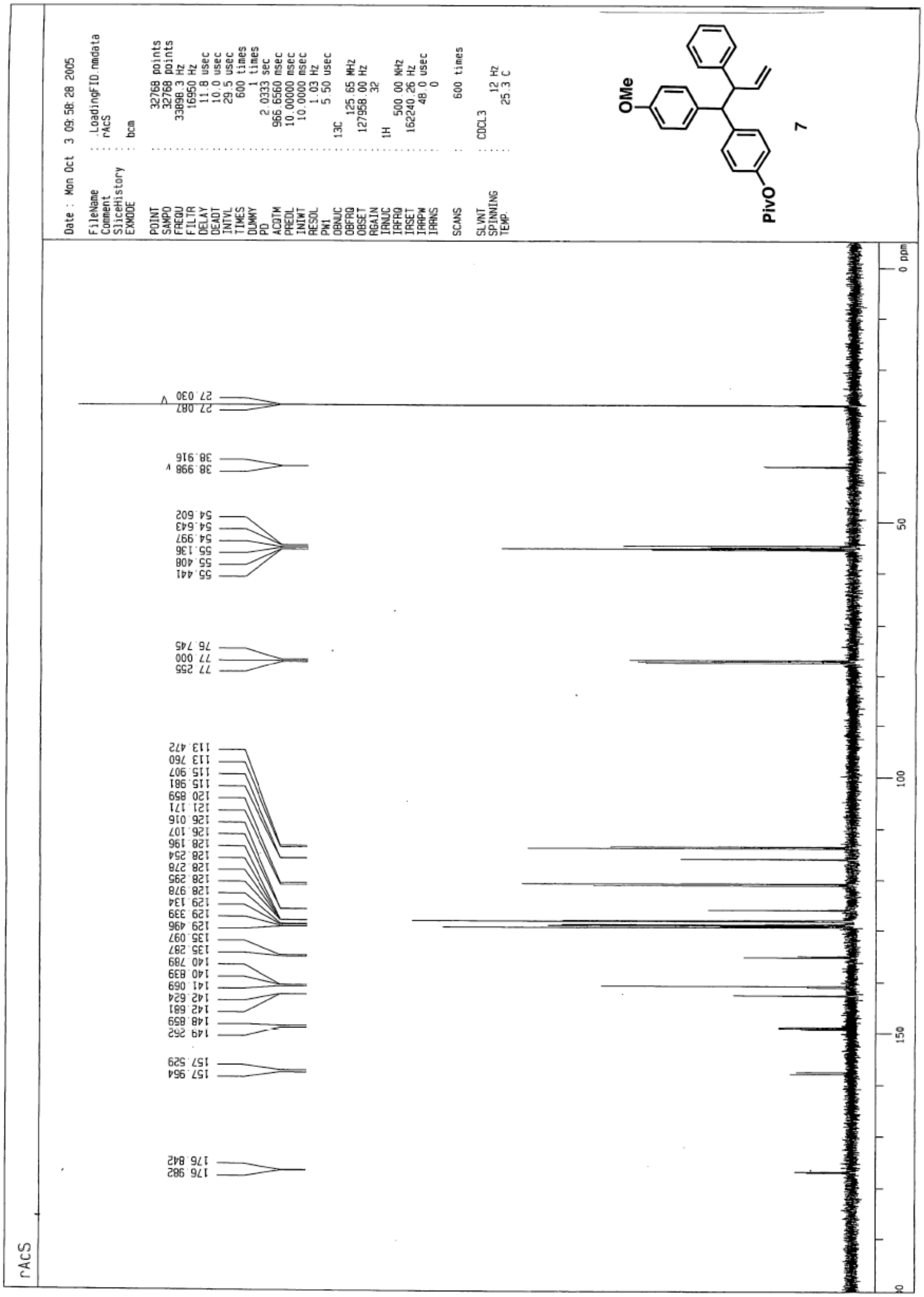
OMe
PivO
7

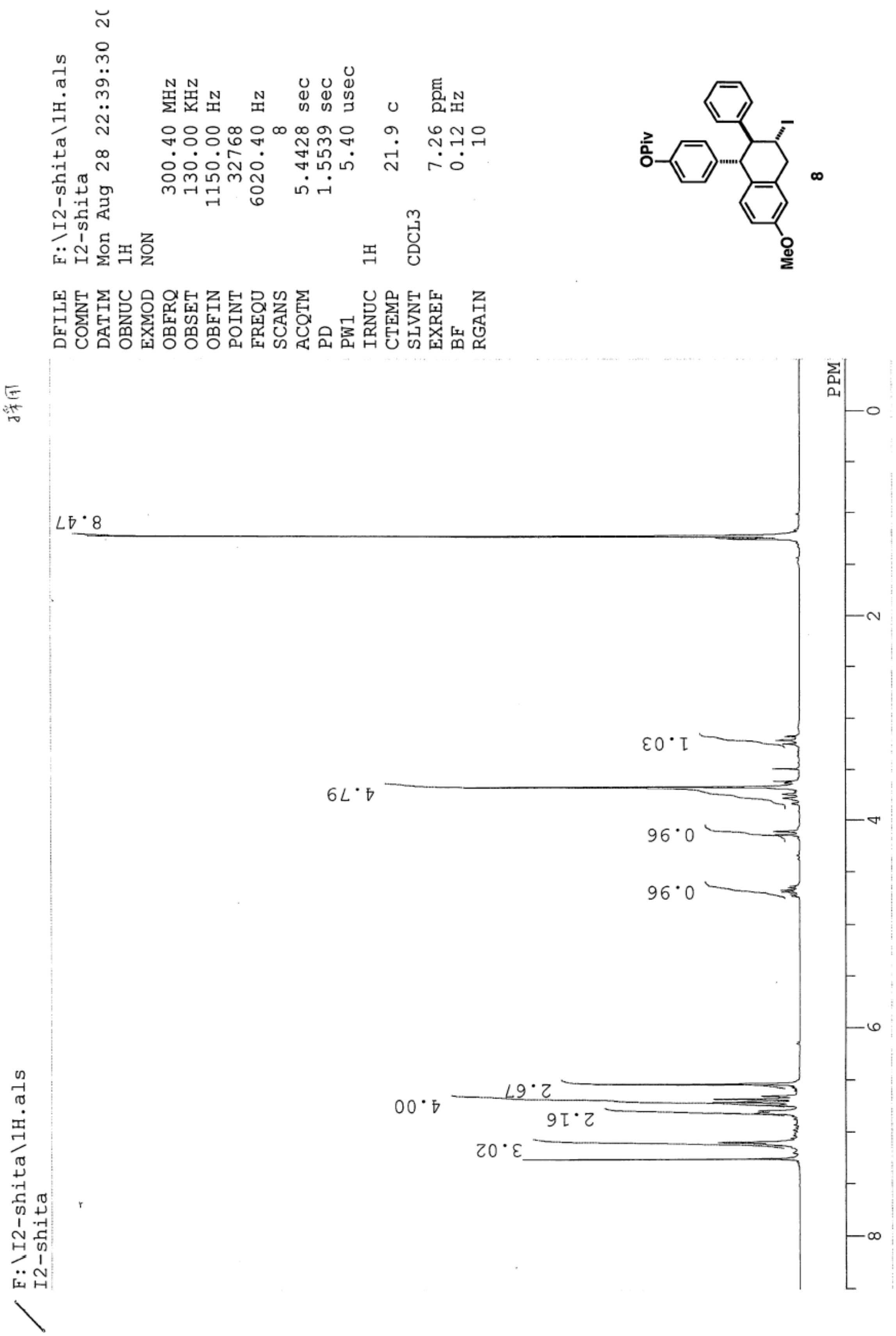
F:\I2-shita\1H.als
I2-shita
DFILE F:\I2-shita\1H.als
COMNT I2-shita
DATIM Mon Aug 28 22:39:30 2(
OBNUC 1H
EXMOD NON
OBFRQ 300.40 MHz
OBSET 130.00 KHz
OBFIN 1150.00 Hz
POINT 32768
FREQU 6020.40 Hz
SCANS 8
ACQTM 5.4428 sec
PD 1.5539 sec
PW1 5.40 usec
IRNUC 1H
CTEMP 21.9 c
SLVNT CDCL3
EXREF 7.26 ppm
BF 0.12 Hz
RGAIN 10
OPiv
MeO
I
8
8.47
1.03
4.79
0.96
0.96
4.00
2.67
2.16
3.02
PPM
0
2
4
6
8

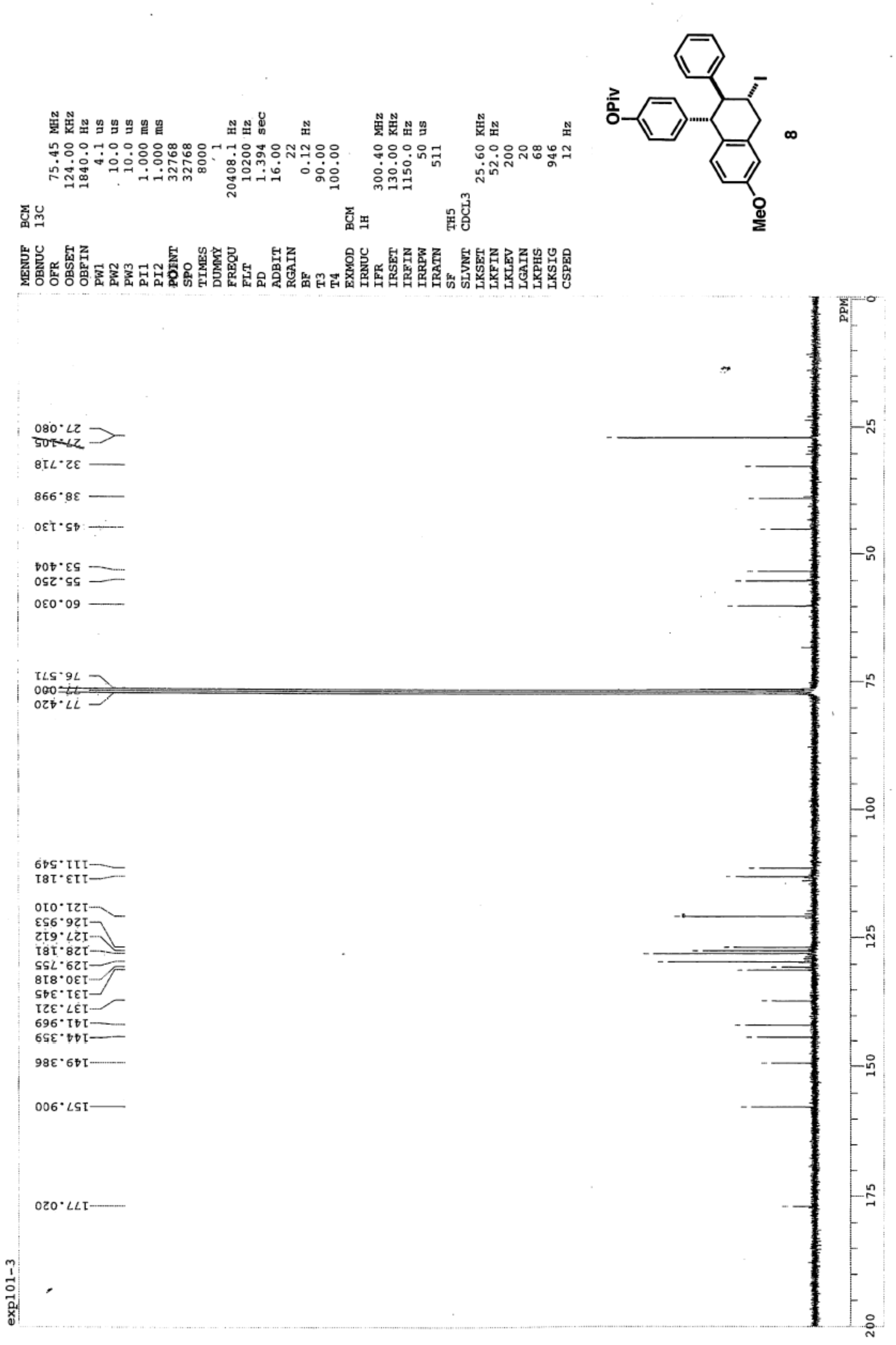

OPiv
MeO
8
MENUF BCM
OBNUC 13C
OFR 75.45 MHz
OBSET 124.00 KHz
OBFIN 1840.0 Hz
PW1 4.1 us
PW2 10.0 us
PW3 10.0 us
PI1 1.000 ms
PI2 1.000 ms
POINT 32768
SPO 32768
TIMES 8000
DUMMY 1
FREQU 20408.1 Hz
FLT 10200 Hz
PD 1.394 sec
ADBIT 16.00
RGAIN 22
BF 0.12 Hz
T3 90.00
T4 100.00
EXMOD BCM
IRNUC 1H
IFR 300.40 MHz
IRSET 130.00 KHz
IRFIN 1150.0 Hz
IRRPW 50 us
IRATN 511
SF TH5
SLVNT CDCL3
LKSET 25.60 KHz
LKFIN 52.0 Hz
LKLEV 200
LGAIN 20
LKPHS 68
LKSIG 946
CSPED 12 Hz
27.080
27.105
32.718
38.998
45.130
53.404
55.250
60.030
76.571
77.000
77.420
111.549
113.181
121.010
126.953
127.612
128.181
129.755
130.818
131.345
137.321
141.969
144.359
149.386
157.900
177.020
exp101-3
PPM
0
25
50
75
100
125
150
175
200

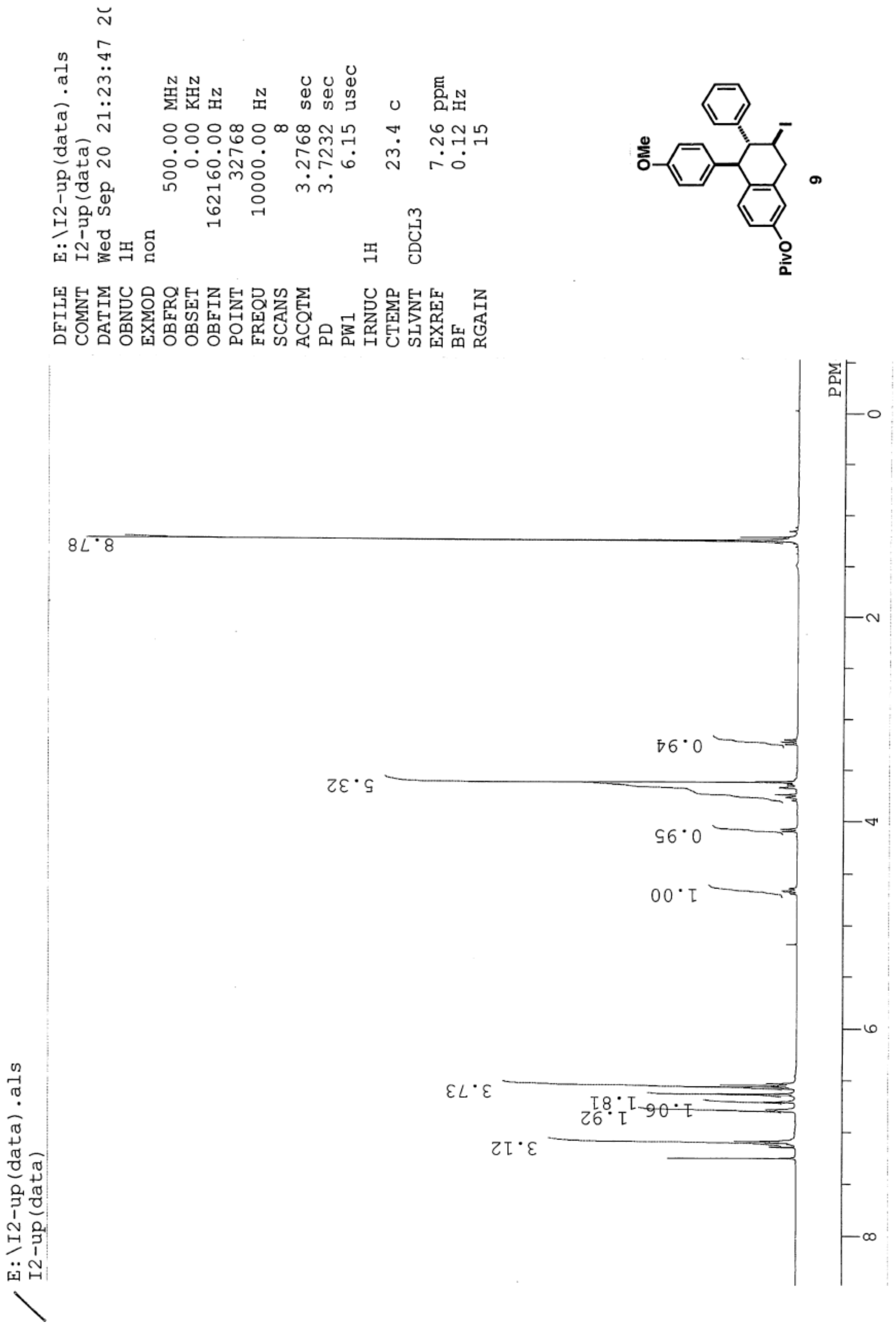
E:\I2-up(data).als
I2-up(data)
DFILE E:\I2-up(data).als
COMNT I2-up(data)
DATIM Wed Sep 20 21:23:47 2(
OBNUC 1H
EXMOD non
OBFRQ 500.00 MHz
OBSET 0.00 KHz
OBFIN 162160.00 Hz
POINT 32768
FREQU 10000.00 Hz
SCANS 8
ACQTM 3.2768 sec
PD 3.7232 sec
PW1 6.15 usec
IRNUC 1H
CTEMP 23.4 c
SLVNT CDCL3
EXREF 7.26 ppm
BF 0.12 Hz
RGAIN 15
OMe
PivO
I
9
8.78
0.94
5.32
0.95
1.00
3.73
1.06
1.81
1.92
3.12
PPM
0
2
4
6
8

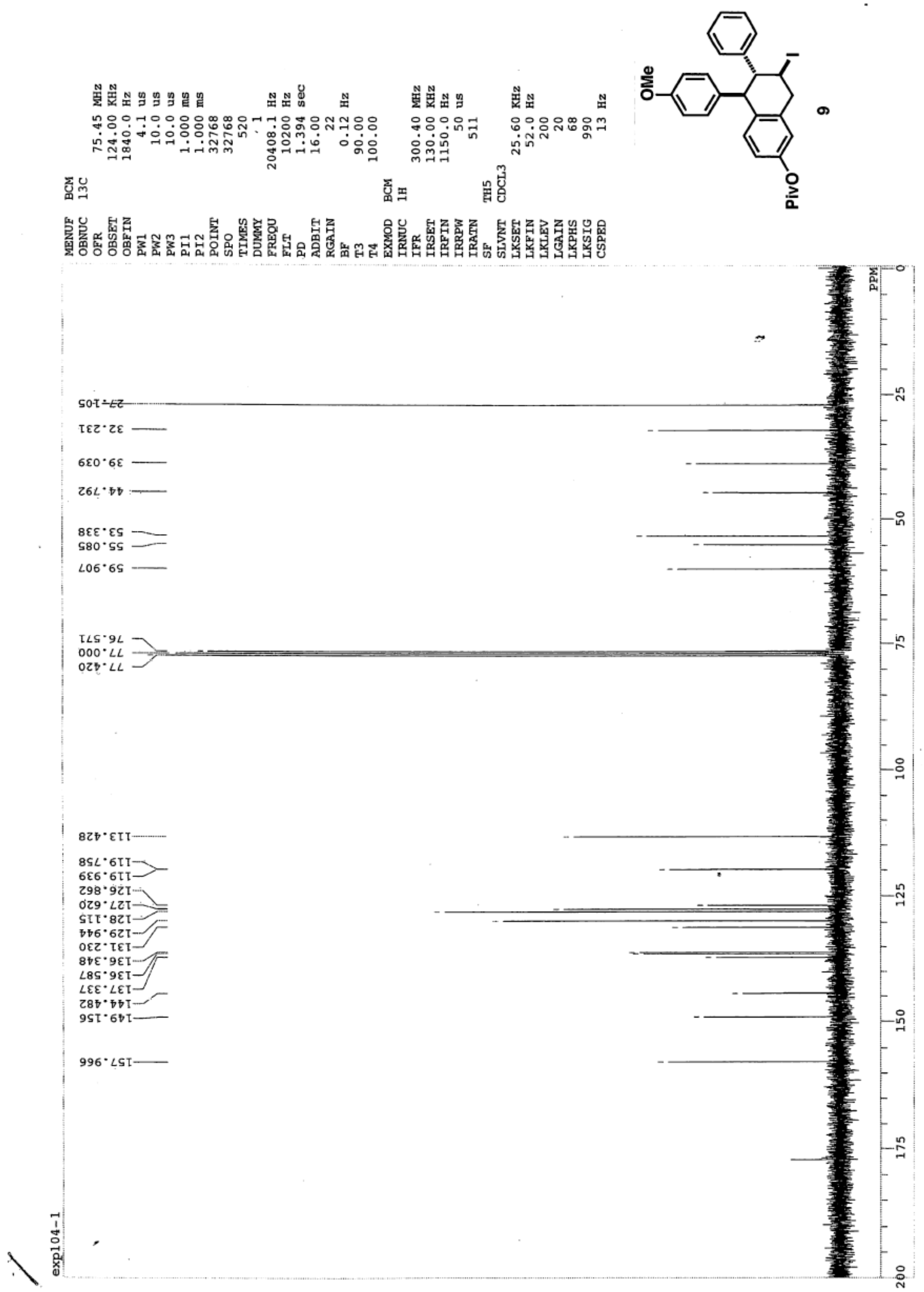
exp104-1
27.105
32.231
39.039
44.792
53.338
55.085
59.907
76.571
77.000
77.420
113.428
119.758
119.939
126.862
127.620
128.115
129.944
131.230
136.348
136.587
137.337
144.482
149.156
157.966
PPM
OMe
PivO
I
9

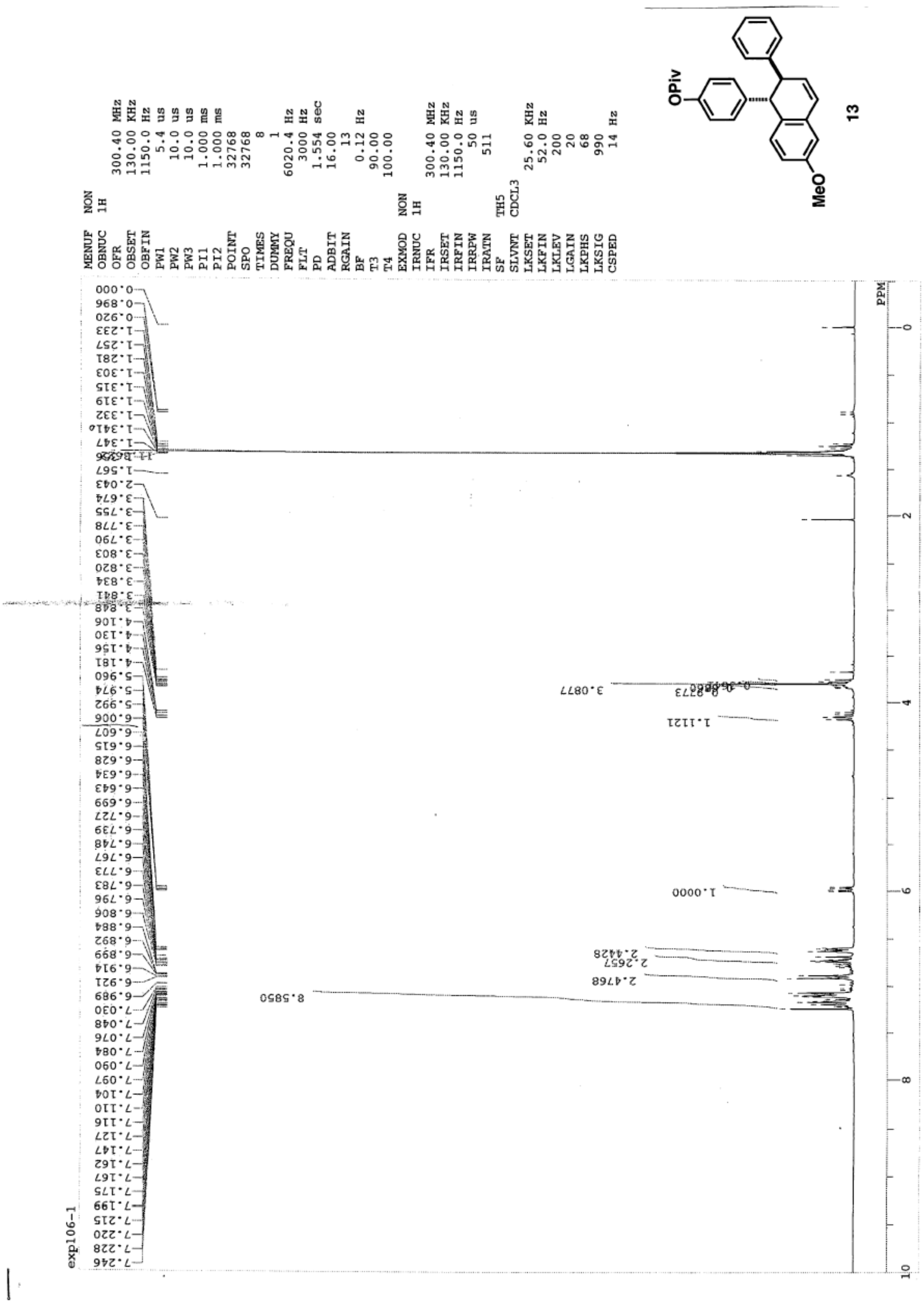
exp106-1
MENUF NON
OBNUC 1H
OFR 300.40 MHz
OBSET 130.00 KHz
OBFIN 1150.0 Hz
PW1 5.4 us
PW2 10.0 us
PW3 10.0 us
PI1 1.000 ms
PI2 1.000 ms
POINT 32768
SPO 32768
TIMES 8
DUMMY 1
FREQU 6020.4 Hz
FLT 3000 Hz
PD 1.554 sec
ADBIT 16.00
RGAIN 13
BF 0.12 Hz
T3 90.00
T4 100.00
EXMOD NON
IRNUC 1H
IFR 300.40 MHz
IRSET 130.00 KHz
IRFIN 1150.0 Hz
IRRPW 50 us
IRATN 511
SF TH5
SLVNT CDCL3
LKSET 25.60 KHz
LKFIN 52.0 Hz
LKLEV 200
LGAIN 20
LKPHS 68
LKSIG 990
CSPED 14 Hz
OPiv
MeO
13
PPM

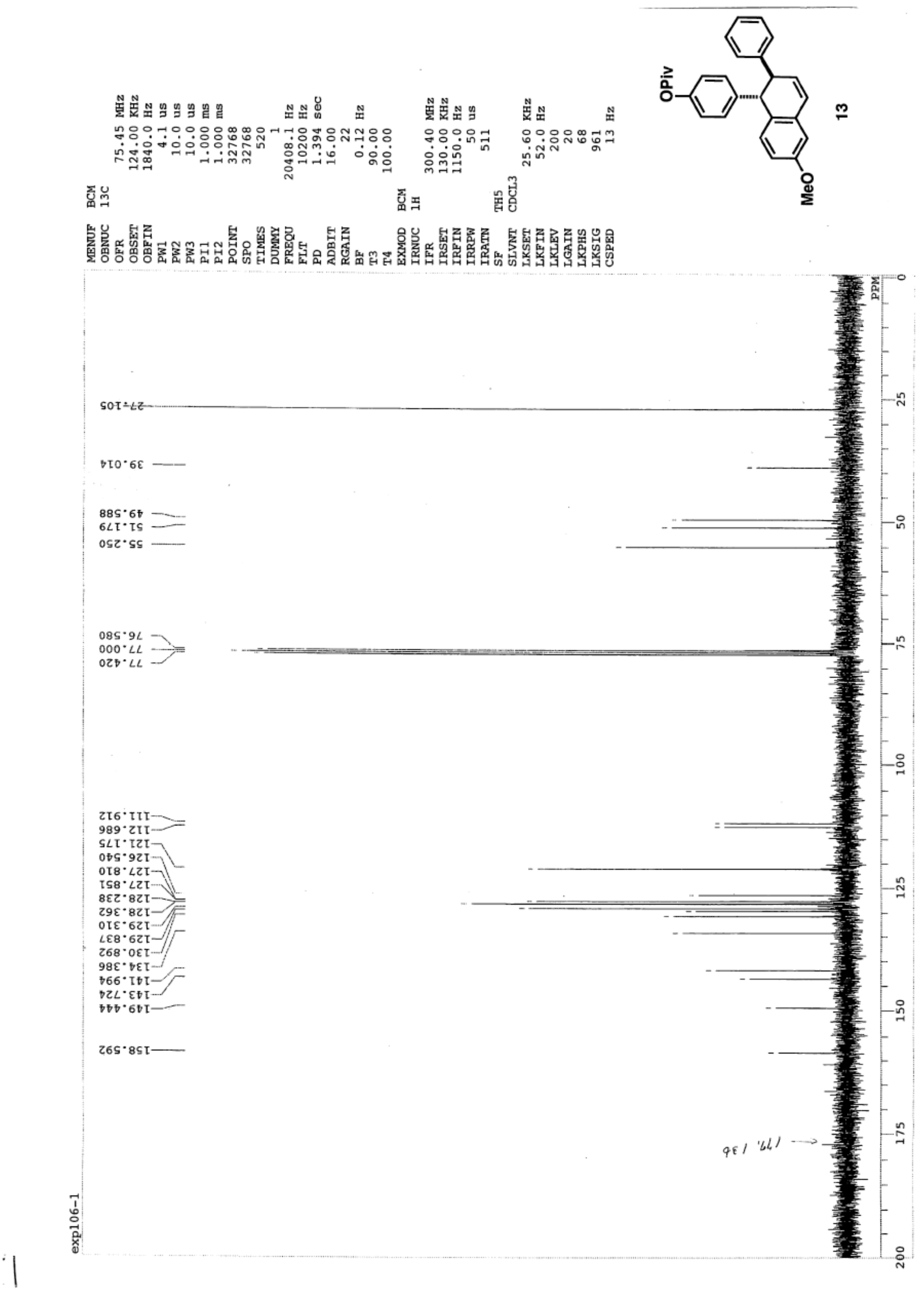
exp106-1
MENUF BCM
OBNUC 13C
OFR 75.45 MHz
OBSET 124.00 KHz
OBFIN 1840.0 Hz
PW1 4.1 us
PW2 10.0 us
PW3 10.0 us
PI1 1.000 ms
PI2 1.000 ms
POINT 32768
SPO 32768
TIMES 520
DUMMY 1
FREQU 20408.1 Hz
FLT 10200 Hz
PD 1.394 sec
ADBIT 16.00
RGAIN 22
BF 0.12 Hz
T3 90.00
T4 100.00
EXMOD BCM
IRNUC 1H
IFR 300.40 MHz
IRSET 130.00 KHz
IRFIN 1150.0 Hz
IRRPW 50 us
IRATN 511
SF TH5
SLVNT CDCL3
LKSET 25.60 KHz
LKFIN 52.0 Hz
LKLEV 200
LGAIN 20
LKPHS 68
LKSIG 961
CSPED 13 Hz
OPiv
MeO
13
27.105
39.014
49.588
51.179
55.250
76.580
77.000
77.420
111.912
112.686
121.175
126.540
127.810
127.851
128.238
128.362
129.310
129.837
130.892
134.386
141.994
143.724
149.444
158.592
177.136
PPM
0
25
50
75
100
125
150
175
200

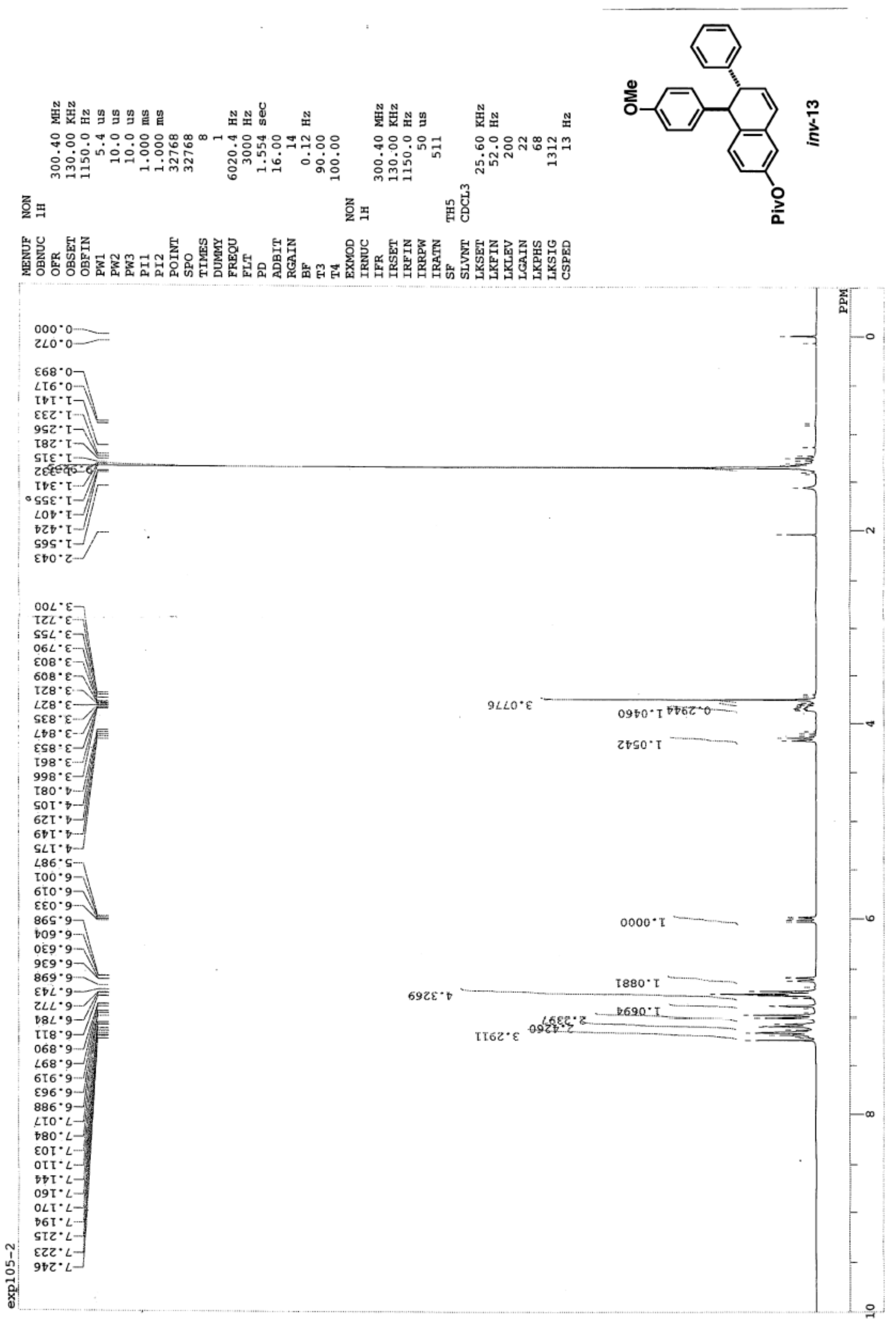

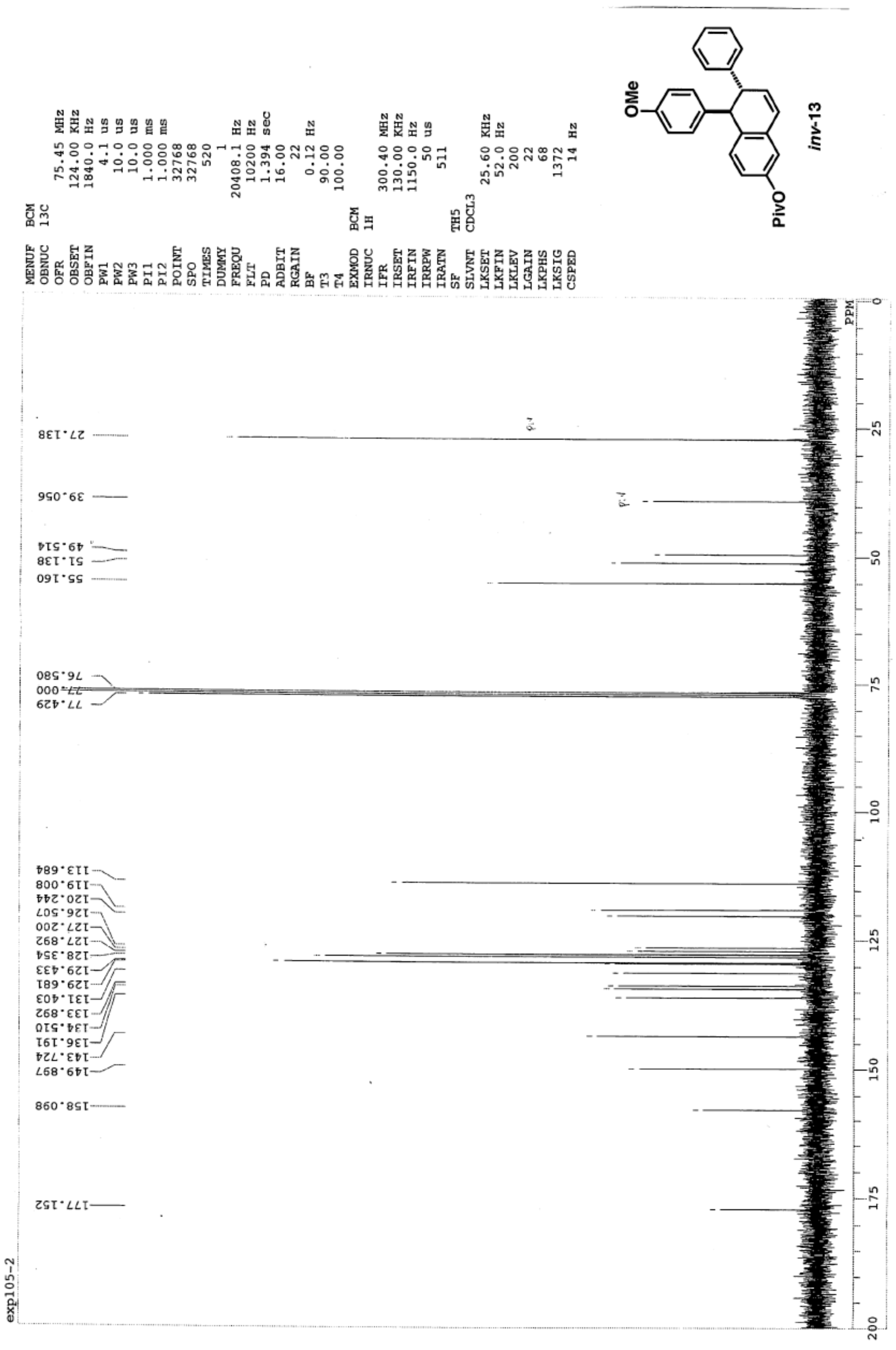

exp105-2
MENUF BCM
OBNUC 13C
OFR 75.45 MHz
OBSET 124.00 KHz
OBFIN 1840.0 Hz
PW1 4.1 us
PW2 10.0 us
PW3 10.0 us
PI1 1.000 ms
PI2 1.000 ms
POINT 32768
SPO 32768
TIMES 520
DUMMY 1
FREQU 20408.1 Hz
FLT 10200 Hz
PD 1.394 sec
ADBIT 16.00
RGAIN 22
BF 0.12 Hz
T3 90.00
T4 100.00
EXMOD BCM
IRNUC 1H
IFR 300.40 MHz
IRSET 130.00 KHz
IRFIN 1150.0 Hz
IRRPW 50 us
IRATN 511
SF TH5
SLVNT CDCL3
LKSET 25.60 KHz
LKFIN 52.0 Hz
LKLEV 200
LGAIN 22
LKPHS 68
LKSIG 1372
CSPED 14 Hz
27.138
39.056
49.514
51.138
55.160
76.580
77.000
77.429
113.684
119.008
120.244
126.507
127.200
127.892
128.354
129.433
129.681
131.403
133.892
134.510
136.191
143.724
149.897
158.098
177.152
PPM
0
25
50
75
100
125
150
175
200
OMe
PivO
inv-13

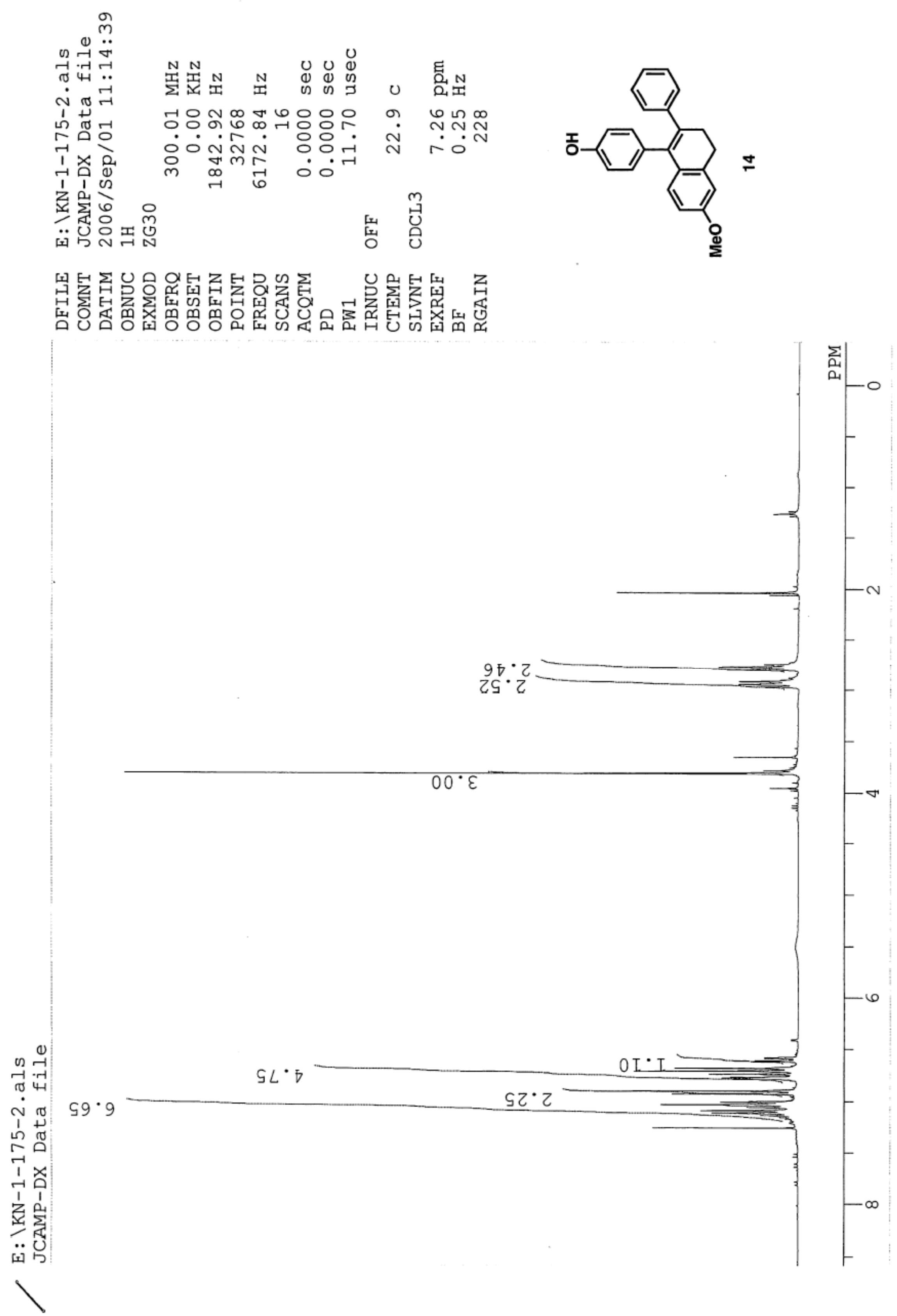
E:\KN-1-175-2.als
JCAMP-DX Data file
DFILE E:\KN-1-175-2.als
COMNT JCAMP-DX Data file
DATIM 2006/Sep/01 11:14:39
OBNUC 1H
EXMOD ZG30
OBFRQ 300.01 MHz
OBSET 0.00 KHz
OBFIN 1842.92 Hz
POINT 32768
FREQU 6172.84 Hz
SCANS 16
ACQTM 0.0000 sec
PD 0.0000 sec
PW1 11.70 usec
IRNUC OFF
CTEMP 22.9 c
SLVNT CDCL3
EXREF 7.26 ppm
BF 0.25 Hz
RGAIN 228
OH
MeO
14
6.65
4.75
2.25
1.10
3.00
2.52
2.46
PPM
0
2
4
6
8

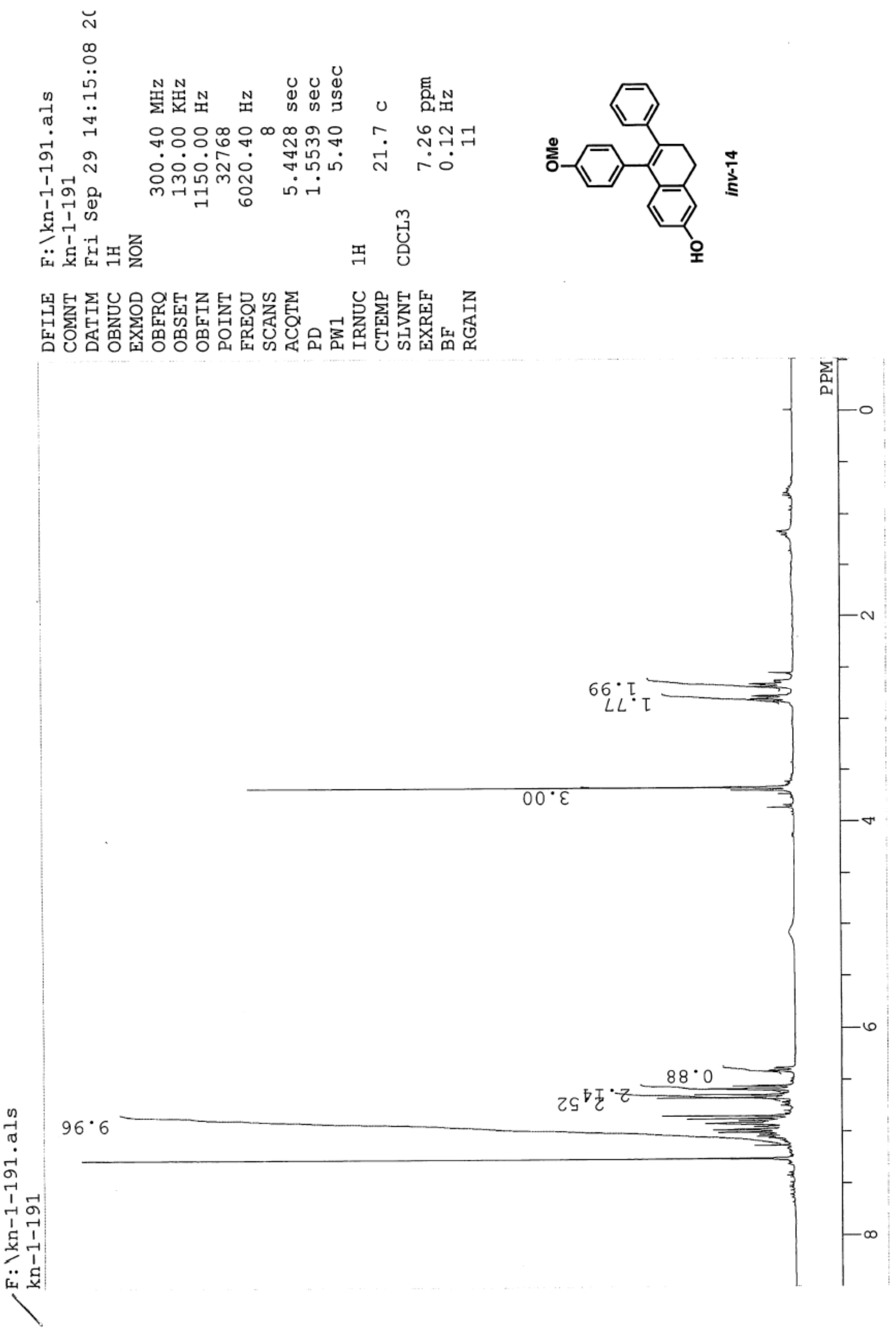
DFILE F:\kn-1-191.als
COMNT kn-1-191
DATIM Fri Sep 29 14:15:08 20
OBNUC 1H
EXMOD NON
OBFRQ 300.40 MHz
OBSET 130.00 KHz
OBFIN 1150.00 Hz
POINT 32768
FREQU 6020.40 Hz
SCANS 8
ACQTM 5.4428 sec
PD 1.5539 sec
PW1 5.40 usec
IRNUC 1H
CTEMP 21.7 c
SLVNT CDCL3
EXREF 7.26 ppm
BF 0.12 Hz
RGAIN 11
OMe
HO
inv-14
F:\kn-1-191.als
kn-1-191
9.96
2.14
2.52
0.88
3.00
1.77
1.99
PPM
0
2
4
6
8

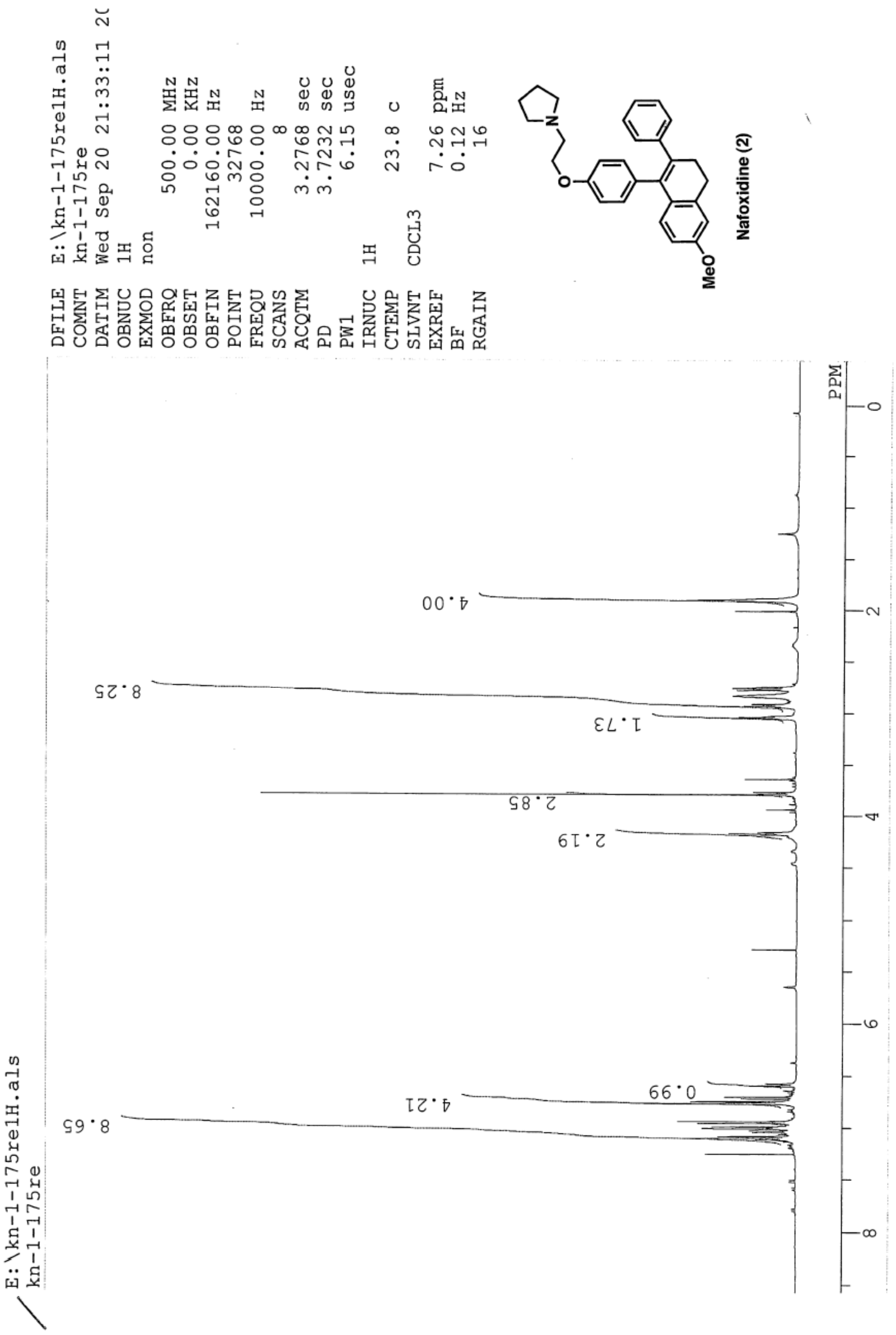

DFILE E:\kn-1-175re1H.als
COMNT kn-1-175re
DATIM Wed Sep 20 21:33:11 2(
OBNUC 1H
EXMOD non
OBFRQ 500.00 MHz
OBSET 0.00 KHz
OBFIN 162160.00 Hz
POINT 32768
FREQU 10000.00 Hz
SCANS 8
ACQTM 3.2768 sec
PD 3.7232 sec
PW1 6.15 usec
IRNUC 1H
CTEMP 23.8 c
SLVNT CDCL3
EXREF 7.26 ppm
BF 0.12 Hz
RGAIN 16
MeO
O
N
Nafoxidine (2)
E:\kn-1-175re1H.als
kn-1-175re
8.65
4.21
0.99
2.19
2.85
1.73
8.25
4.00
PPM
0
2
4
6
8

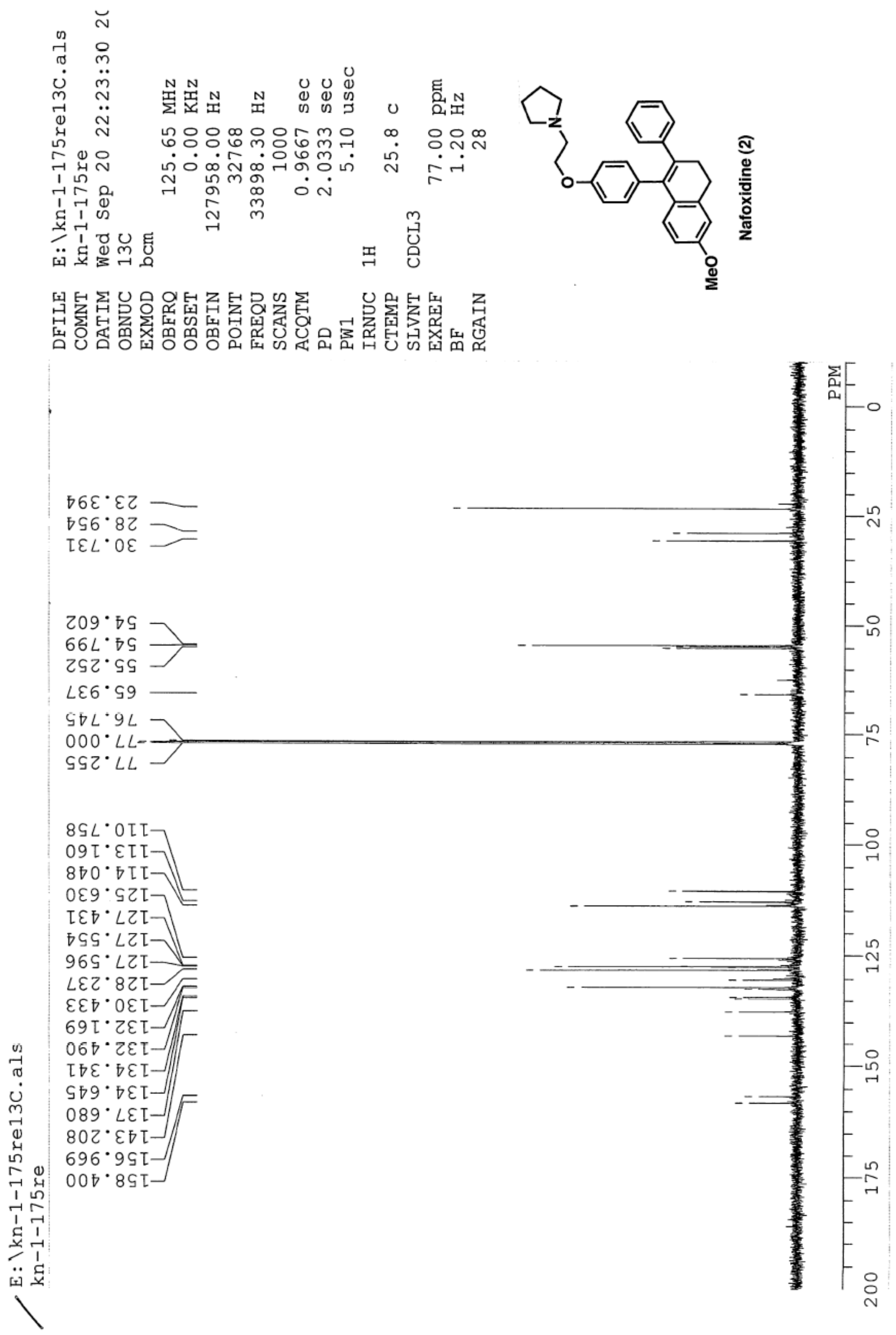
E:\kn-1-175re13C.als
kn-1-175re
DFILE E:\kn-1-175re13C.als
COMNT kn-1-175re
DATIM Wed Sep 20 22:23:30 20
OBNUC 13C
EXMOD bcm
OBFRQ 125.65 MHz
OBSET 0.00 KHz
OBFIN 127958.00 Hz
POINT 32768
FREQU 33898.30 Hz
SCANS 1000
ACQTM 0.9667 sec
PD 2.0333 sec
PW1 5.10 usec
IRNUC 1H
CTEMP 25.8 c
SLVNT CDCL3
EXREF 77.00 ppm
BF 1.20 Hz
RGAIN 28
23.394
28.954
30.731
54.602
54.799
55.252
65.937
76.745
77.000
77.255
110.758
113.160
114.048
125.630
127.431
127.554
127.596
128.237
130.433
132.169
132.490
134.341
134.645
137.680
143.208
156.969
158.400
MeO
O
N
Nafoxidine (2)
PPM
0
25
50
75
100
125
150
175
200

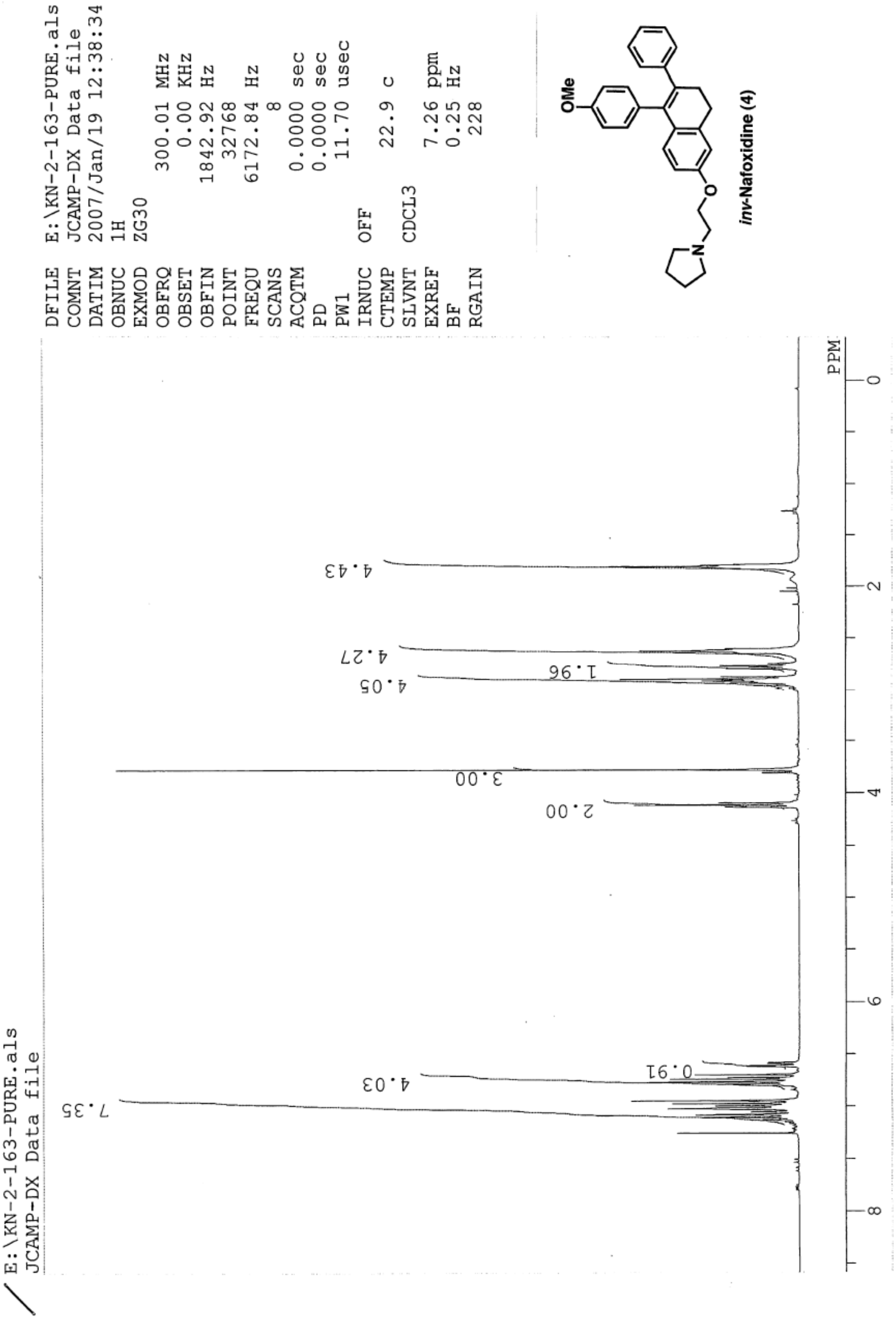
E:\KN-2-163-PURE.als
JCAMP-DX Data file
DFILE E:\KN-2-163-PURE.als
COMNT JCAMP-DX Data file
DATIM 2007/Jan/19 12:38:34
OBNUC 1H
EXMOD ZG30
OBFRQ 300.01 MHz
OBSET 0.00 KHz
OBFIN 1842.92 Hz
POINT 32768
FREQU 6172.84 Hz
SCANS 8
ACQTM 0.0000 sec
PD 0.0000 sec
PW1 11.70 usec
IRNUC OFF
CTEMP 22.9 c
SLVNT CDCL3
EXREF 7.26 ppm
BF 0.25 Hz
RGAIN 228
OMe
O
N
inv-Nafoxidine (4)
7.35
4.03
0.91
2.00
3.00
4.05
1.96
4.27
4.43
PPM
0
2
4
6
8

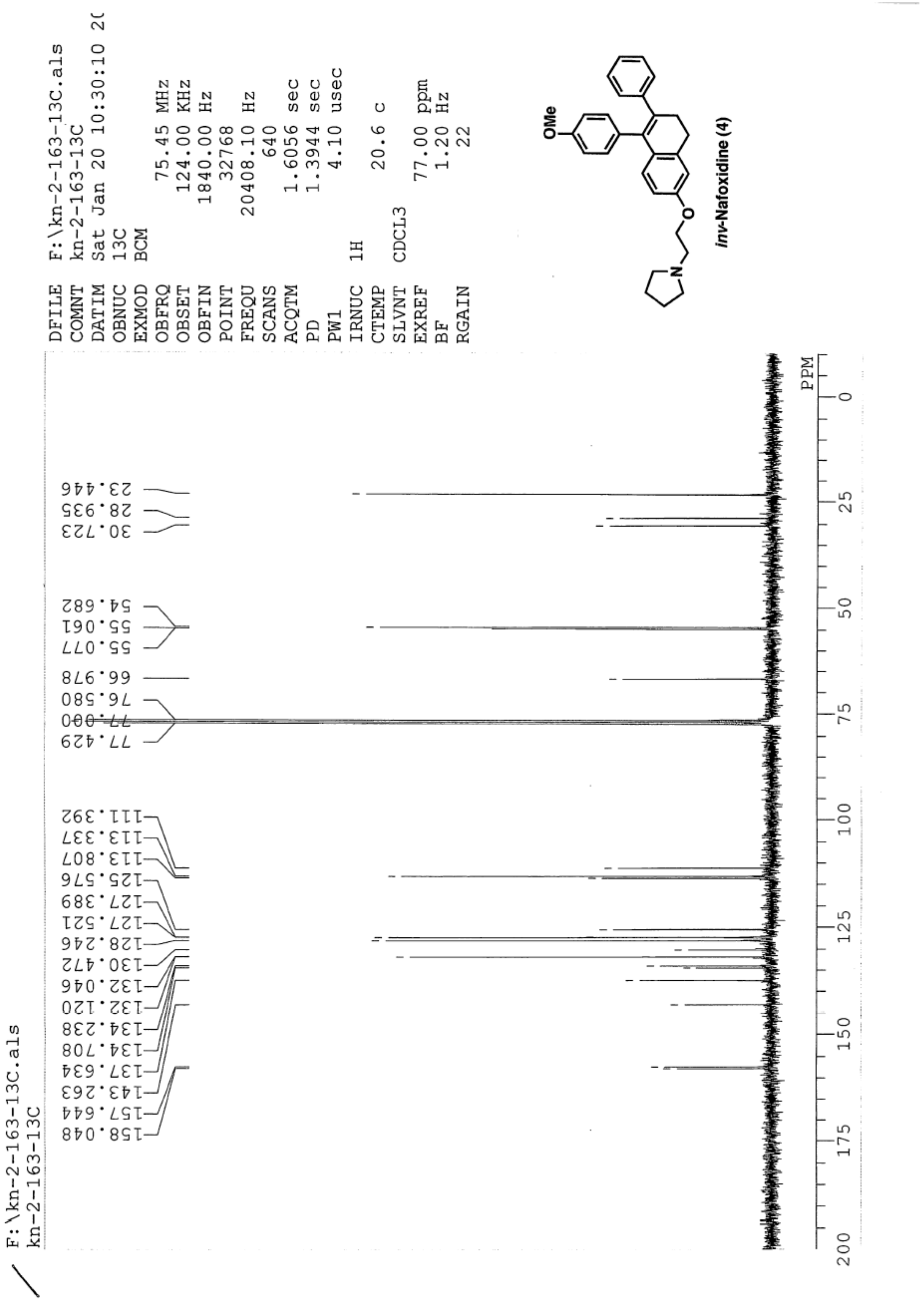


*inv*-Nafoxidine (4)

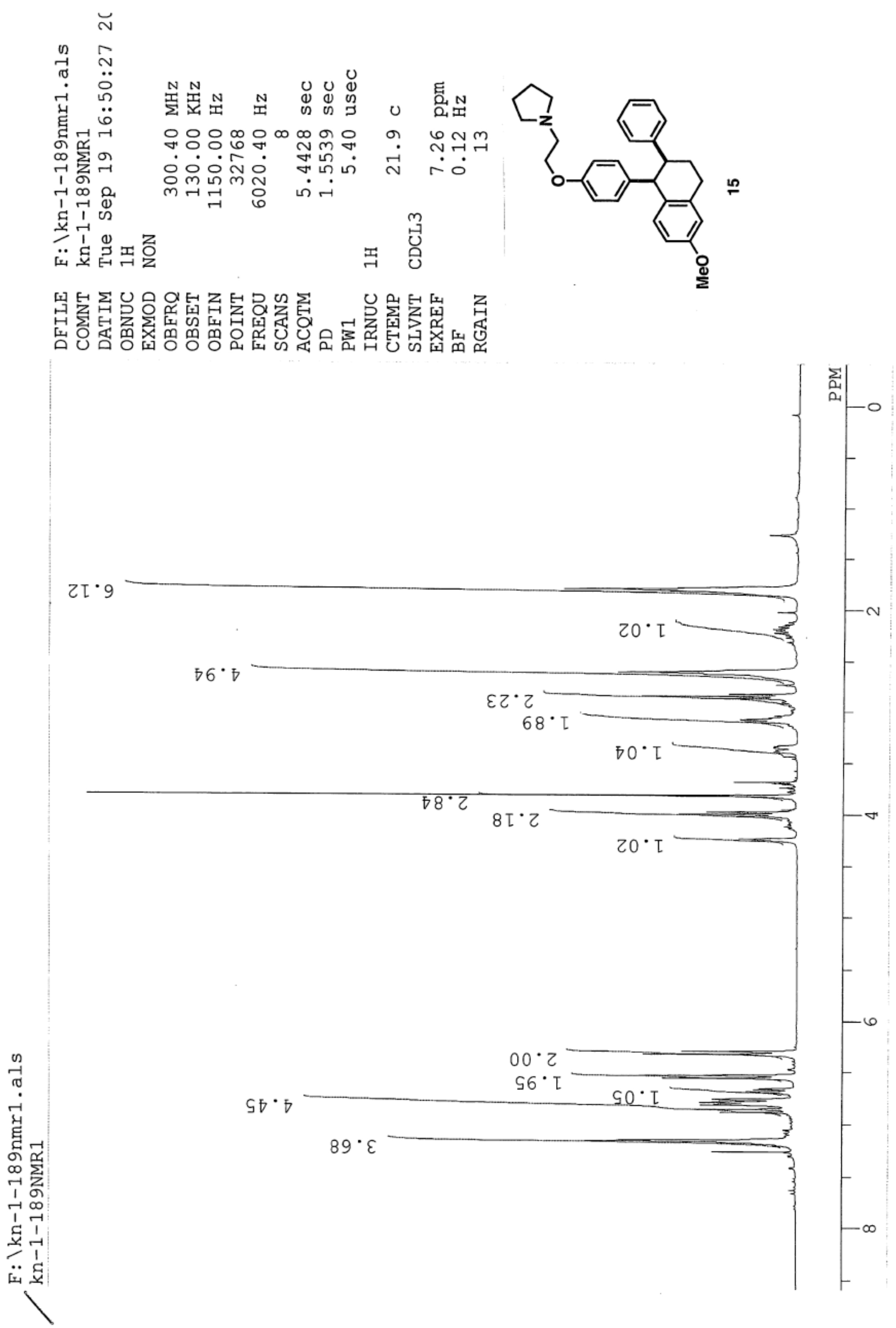
F:\kn-1-189nmr1.als
kn-1-189NMR1
DFILE F:\kn-1-189nmr1.als
COMNT kn-1-189NMR1
DATIM Tue Sep 19 16:50:27 2(
OBNUC 1H
EXMOD NON
OBFRQ 300.40 MHz
OBSET 130.00 KHz
OBFIN 1150.00 Hz
POINT 32768
FREQU 6020.40 Hz
SCANS 8
ACQTM 5.4428 sec
PD 1.5539 sec
PW1 5.40 usec
IRNUC 1H
CTEMP 21.9 c
SLVNT CDCL3
EXREF 7.26 ppm
BF 0.12 Hz
RGAIN 13
MeO
O
N
15
6.12
1.02
4.94
2.23
1.89
1.04
2.84
2.18
1.02
2.00
1.95
1.05
4.45
3.68
PPM
0
2
4
6
8

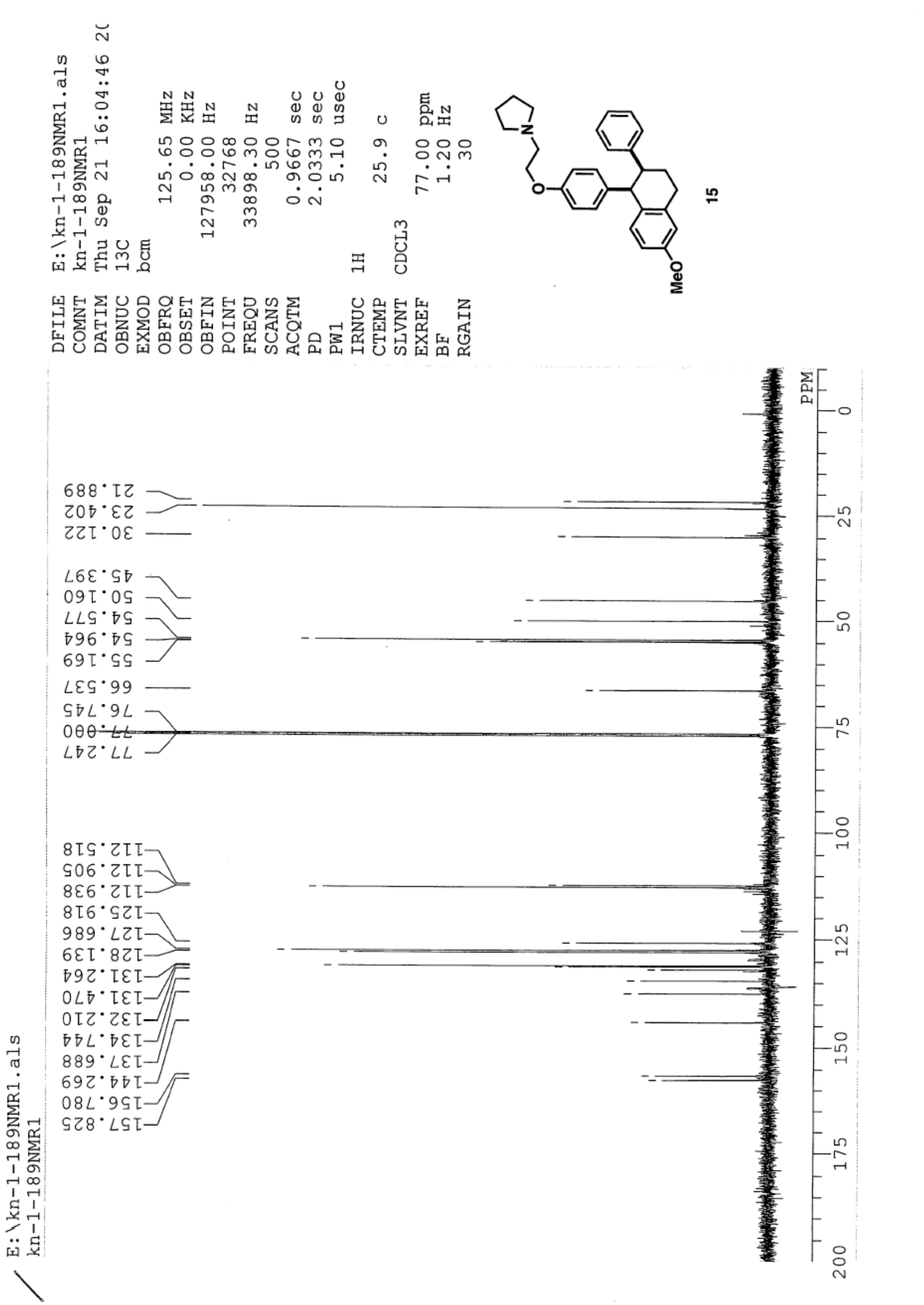
E:\kn-1-189NMR1.als
kn-1-189NMR1
DFILE E:\kn-1-189NMR1.als
COMNT kn-1-189NMR1
DATIM Thu Sep 21 16:04:46 2(
OBNUC 13C
EXMOD bcm
OBFRQ 125.65 MHz
OBSET 0.00 KHz
OBFIN 127958.00 Hz
POINT 32768
FREQU 33898.30 Hz
SCANS 500
ACQTM 0.9667 sec
PD 2.0333 sec
PW1 5.10 usec
IRNUC 1H
CTEMP 25.9 c
SLVNT CDCL3
EXREF 77.00 ppm
BF 1.20 Hz
RGAIN 30
21.889
23.402
30.122
45.397
50.160
54.577
54.964
55.169
66.537
76.745
77.000
77.247
112.518
112.905
112.938
125.918
127.686
128.139
131.264
131.470
132.210
134.744
137.688
144.269
156.780
157.825
PPM
0
25
50
75
100
125
150
175
200
MeO
O
N
15

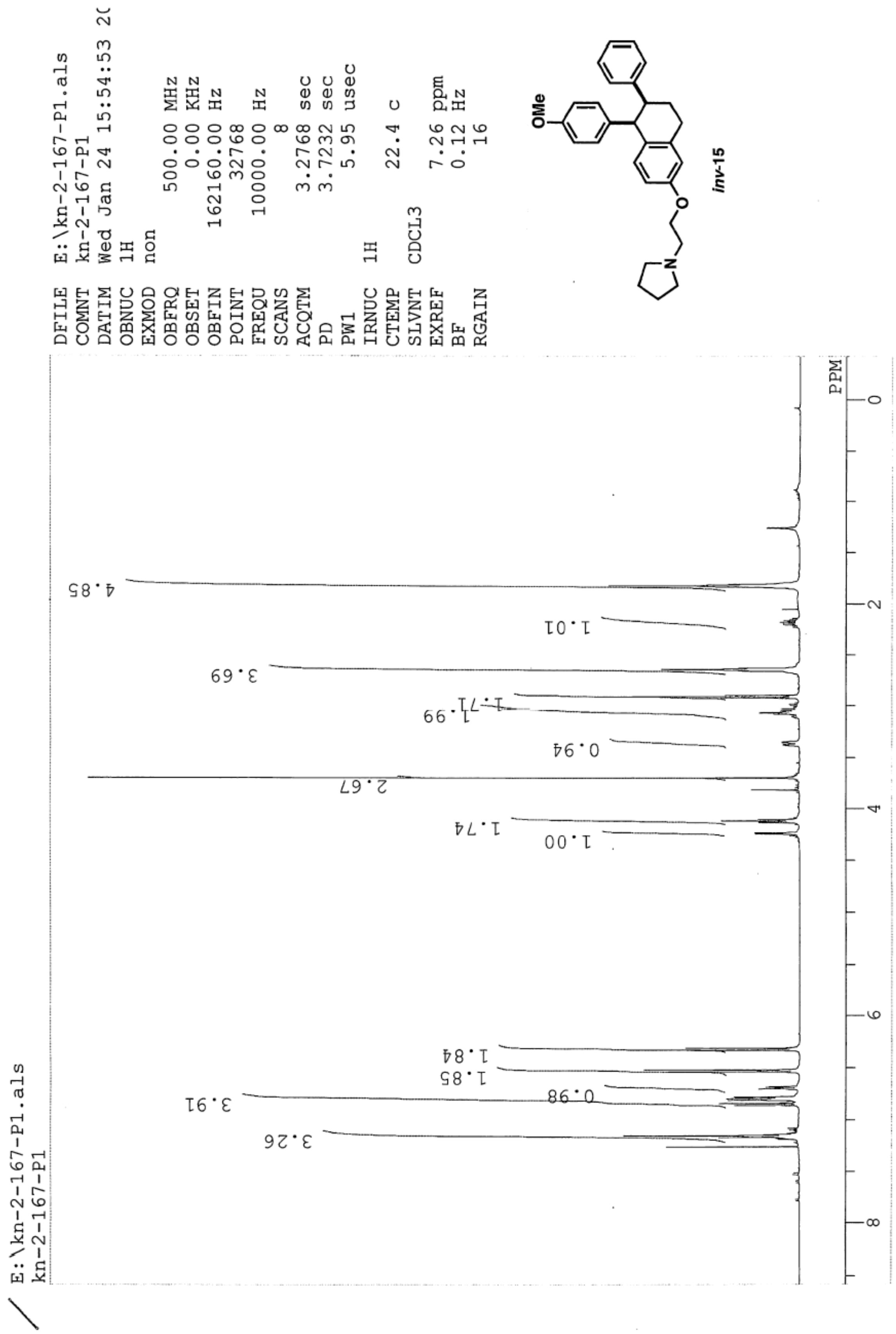
DFILE E:\kn-2-167-P1.als
COMNT kn-2-167-P1
DATIM Wed Jan 24 15:54:53 2(
OBNUC 1H
EXMOD non
OBFRQ 500.00 MHz
OBSET 0.00 KHz
OBFIN 162160.00 Hz
POINT 32768
FREQU 10000.00 Hz
SCANS 8
ACQTM 3.2768 sec
PD 3.7232 sec
PW1 5.95 usec
IRNUC 1H
CTEMP 22.4 c
SLVNT CDCL3
EXREF 7.26 ppm
BF 0.12 Hz
RGAIN 16
OMe
N
O
inv-15
E:\kn-2-167-P1.als
kn-2-167-P1
4.85
1.01
3.69
1.71
1.99
0.94
2.67
1.74
1.00
1.84
1.85
0.98
3.91
3.26
PPM
0
2
4
6
8

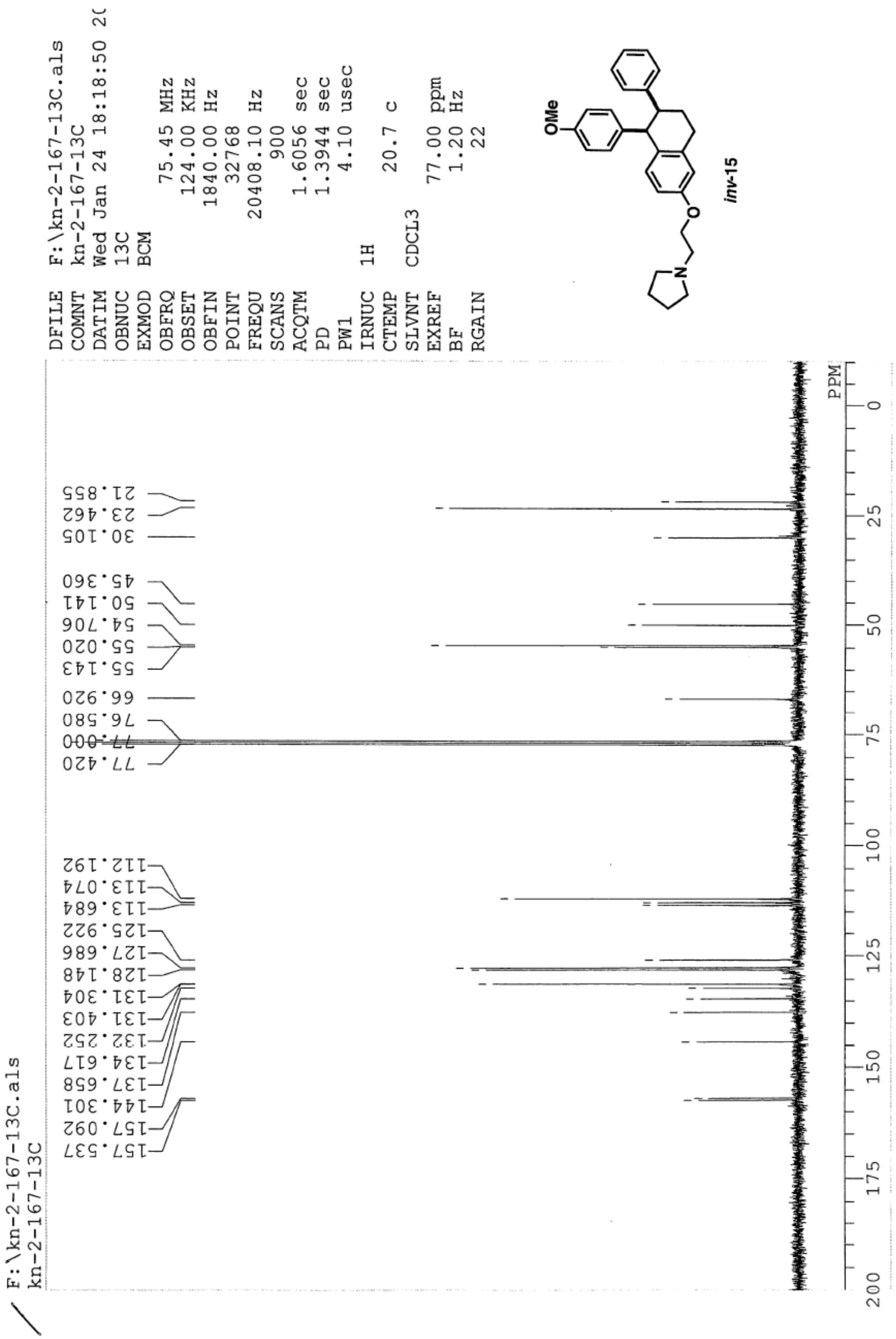
F:\kn-2-167-13C.als
kn-2-167-13C
DFILE F:\kn-2-167-13C.als
COMNT kn-2-167-13C
DATIM Wed Jan 24 18:18:50 2(
OBNUC 13C
EXMOD BCM
OBFRQ 75.45 MHz
OBSET 124.00 KHz
OBFIN 1840.00 Hz
POINT 32768
FREQU 20408.10 Hz
SCANS 900
ACQTM 1.6056 sec
PD 1.3944 sec
PW1 4.10 usec
IRNUC 1H
CTEMP 20.7 c
SLVNT CDCL3
EXREF 77.00 ppm
BF 1.20 Hz
RGAIN 22
21.855
23.462
30.105
45.360
50.141
54.706
55.020
55.143
66.920
76.580
77.000
77.420
112.192
113.074
113.684
125.922
127.686
128.148
131.304
131.403
132.252
134.617
137.658
144.301
157.092
157.537
OMe
N
O
inv-15
PPM
0
25
50
75
100
125
150
175
200

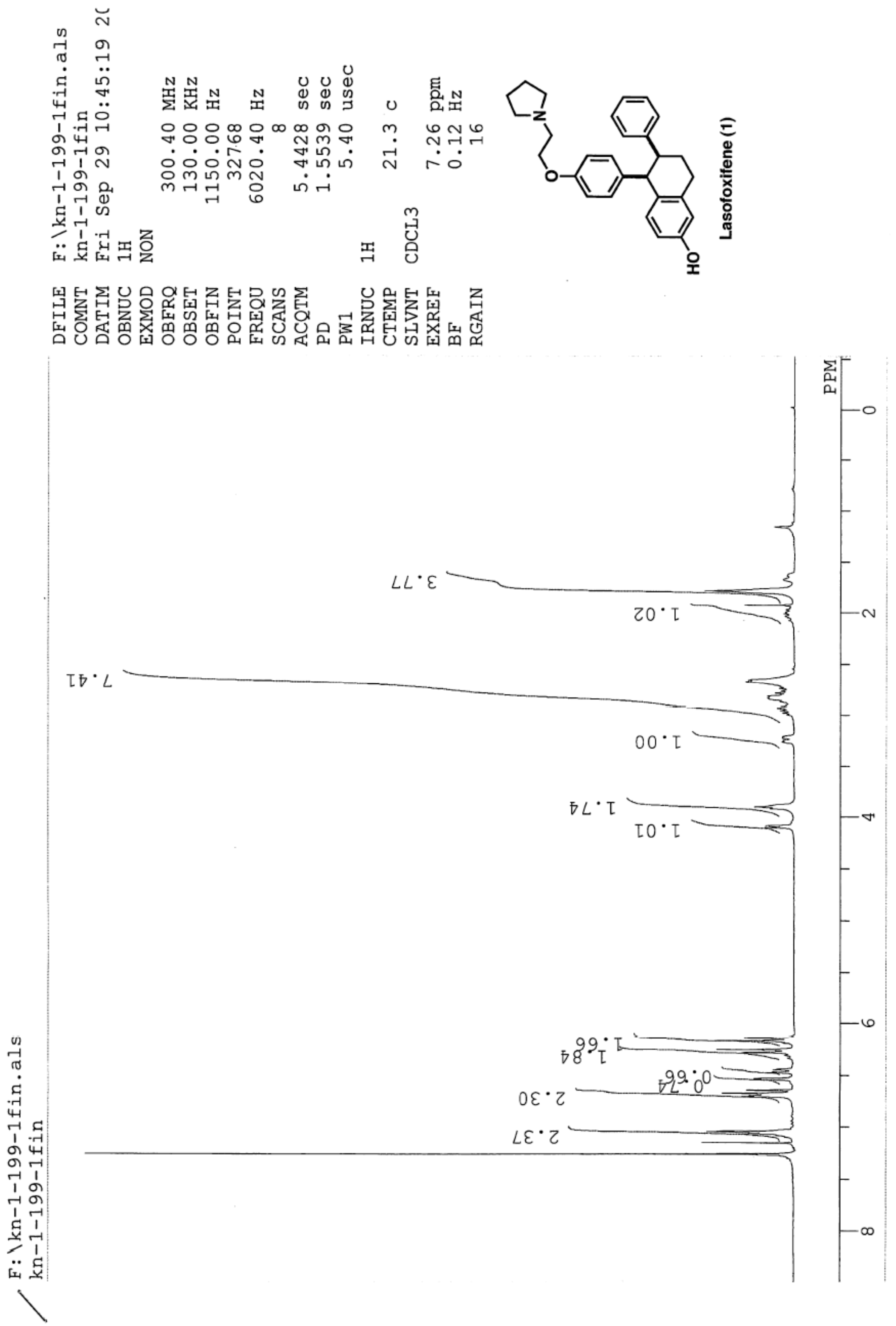
DFILE F:\kn-1-199-1fin.als
COMNT kn-1-199-1fin
DATIM Fri Sep 29 10:45:19 2(
OBNUC 1H
EXMOD NON
OBFRQ 300.40 MHz
OBSET 130.00 KHz
OBFIN 1150.00 Hz
POINT 32768
FREQU 6020.40 Hz
SCANS 8
ACQTM 5.4428 sec
PD 1.5539 sec
PW1 5.40 usec
IRNUC 1H
CTEMP 21.3 c
SLVNT CDCL3
EXREF 7.26 ppm
BF 0.12 Hz
RGAIN 16
Lasofoxifene (1)
F:\kn-1-199-1fin.als
kn-1-199-1fin
PPM

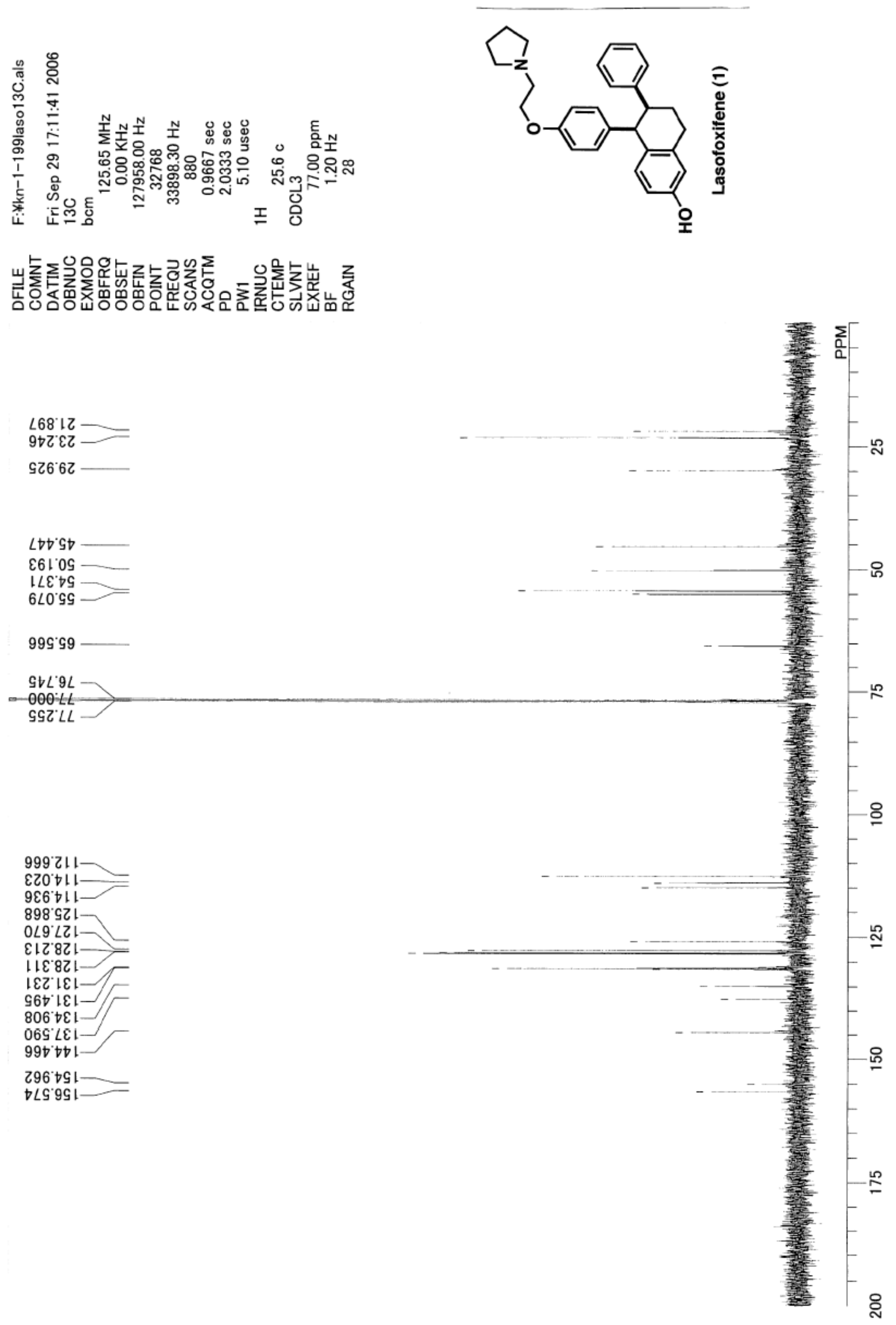
DFILE F:¥kn-1-199laso13C.als
COMNT Fri Sep 29 17:11:41 2006
DATIM 13C
OBNUC bcm
EXMOD 125.65 MHz
OBFRQ 0.00 KHz
OBSET 127958.00 Hz
OBFIN 32768
POINT 33898.30 Hz
FREQU 880
SCANS 0.9667 sec
ACQTM 2.0333 sec
PD 5.10 usec
PW1 1H
IRNUC 25.6 c
CTEMP CDCL3
SLVNT 77.00 ppm
EXREF 1.20 Hz
BF 28
RGAIN
21.897
23.246
29.925
45.447
50.193
54.371
55.079
65.566
76.745
77.000
77.255
112.666
114.023
114.936
125.868
127.670
128.213
128.311
131.231
131.495
134.908
137.590
144.466
154.962
156.574
PPM
25
50
75
100
125
150
175
200
HO
O
N
Lasofoxifene (1)

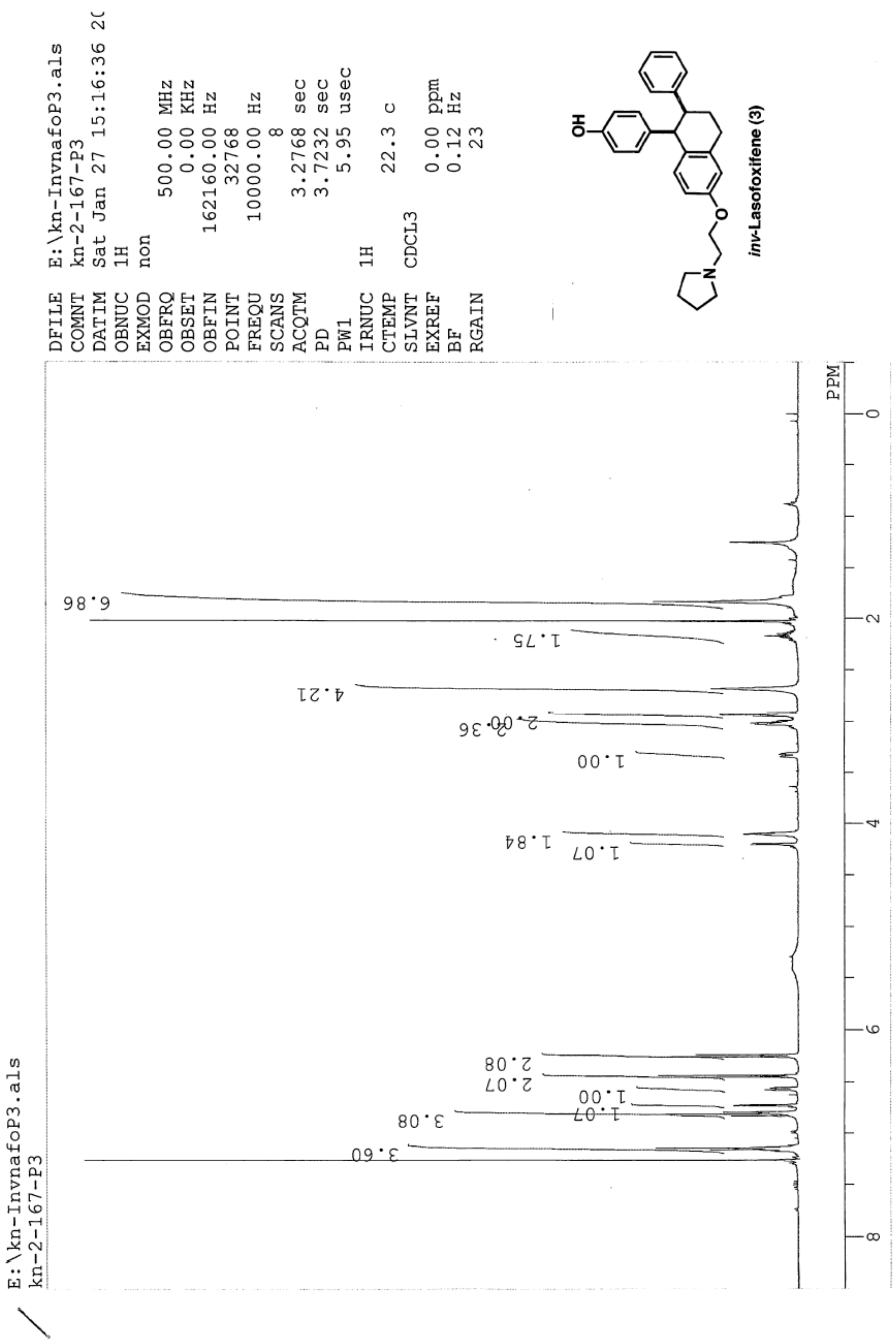

*inv*-Lasofoxifene (3)

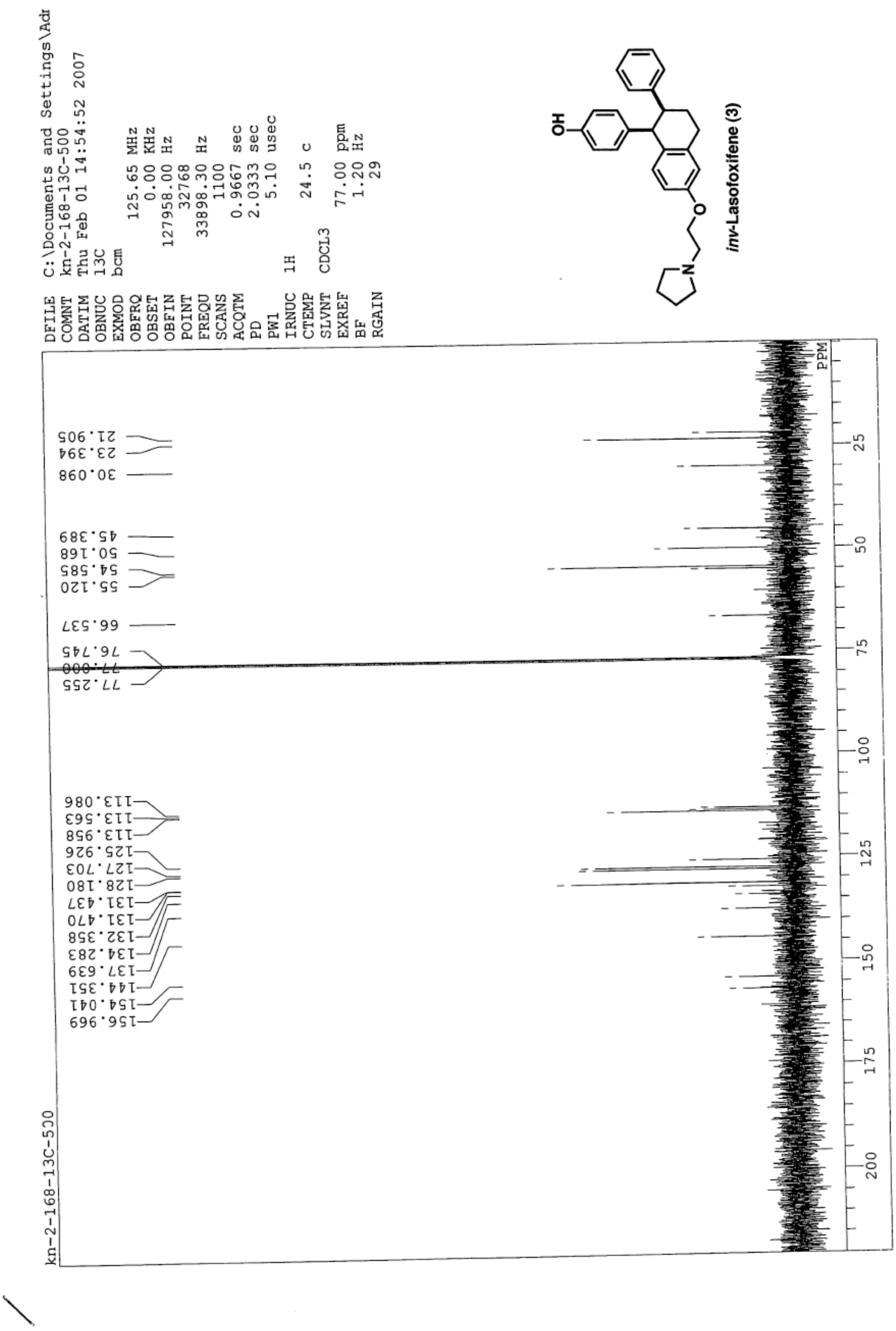
DFILE C:\Documents and Settings\Adr
COMNT kn-2-168-13C-500
DATIM Thu Feb 01 14:54:52 2007
OBNUC 13C
EXMOD bcm
OBFRQ 125.65 MHz
OBSET 0.00 KHz
OBFIN 127958.00 Hz
POINT 32768
FREQU 33898.30 Hz
SCANS 1100
ACQTM 0.9667 sec
PD 2.0333 sec
PW1 5.10 usec
IRNUC 1H
CTEMP 24.5 c
SLVNT CDCL3
EXREF 77.00 ppm
BF 1.20 Hz
RGAIN 29
OH
N
O
*inv*-Lasofoxifene (3)
kn-2-168-13C-500
21.905
23.394
30.098
45.389
50.168
54.585
55.120
66.537
76.745
77.000
77.255
113.086
113.563
113.958
125.926
127.703
128.180
131.437
131.470
132.358
134.283
137.639
144.351
154.041
156.969
PPM
25
50
75
100
125
150
175
200
